# Deciphering signalling network in broad spectrum Near Isogenic Lines of rice resistant to *Magnaporthe oryzae*

**DOI:** 10.1038/s41598-019-50990-8

**Published:** 2019-11-15

**Authors:** Priyanka Jain, Himanshu Dubey, Pankaj Kumar Singh, Amolkumar U. Solanke, Ashok K. Singh, T. R. Sharma

**Affiliations:** 1 ICAR-National Institute for Plant Biotechnology, Pusa Campus, New Delhi, 110012 India; 2 National Agri-Food Biotechnology Institute, Mohali, Punjab India; 3 ICAR-Indian Agricultural Research Institute, New Delhi, 110012 India

**Keywords:** Gene regulatory networks, Gene regulatory networks, Biotic, Biotic

## Abstract

Disease resistance (*R*) genes like *Pi9, Pita, Pi21, Pi54* are playing important role for broad spectrum blast resistance in rice. Development of near isogenic lines (NILs) using these type of broad spectrum genes and understanding their signalling networks is essential to cope up with highly evolving *Magnaporthe oryzae* strains for longer duration. Here, transcriptional-level changes were studied in three near-isogenic lines (PB1 + *Pi1*, PB1 + *Pi9* and PB1 + *Pi54*) of rice resistant to blast infection, to find the loci that are unique to resistant lines developed in the background of Pusa Basmati 1 (PB1). The pathway analysis of loci, unique to resistant NILs compared to susceptible control revealed that plant secondary metabolite synthesis was the common mechanism among all NILs to counter against *M. oryzae* infection. Comparative transcriptome analysis helped to find out common clusters of co-expressed significant differentially expressed loci (SDEL) in both PB1 + *Pi9* and PB1 + *Pi54* NILs. SDELs from these clusters were involved in the synthesis and degradation of starch; synthesis and elongation of fatty acids; hydrolysis of phospholipids; synthesis of phenylpropanoid; and metabolism of ethylene and jasmonic acid. Through detailed analysis of loci specific to each resistant NIL, we identified a network of signalling pathways mediated by each blast resistance gene. The study also offers insights into transcriptomic dynamics, points to a set of important candidate genes that serve as module to regulate the changes in resistant NILs. We suggest that pyramiding of the blast resistance gene *Pi9* with *Pi54* will lead to maximum broad spectrum resistance to *M. oryzae*.

## Introduction

The blast disease of rice, caused by *Magnaporthe oryzae*, is known to lower rice production substantially^1^. If conditions are favourable, the pathogen infects the crop from the seedling stage to the grain-filling stage. Overall losses due to blast are higher in northern India resulting lower yield as well as the inferior quality of grains^2^. The disease is controlled by using resistant lines^3,4^ and applying fungicides. However, host resistance can break down over time as a new strain of pathogens develops, while fungicides can pollute the environment^5^. Breakdown of race specific *R* gene is a common phenomenon due to the highly variable nature of the pathogens. In such situations broad spectrum resistance (*R*) gene play important role to increase the durability of resistance in the management of rice blast disease.

Major changes occur in gene expression when a plant is attacked by a pathogen, and these changes are mostly the results of transcriptional reprogramming. Detailed and systematic analysis of transcriptomes of the near isogenic lines (NILs) helps to understand the mechanism of disease resistance because these lines differ only for single *R* gene^6^. Genes involved in immunity are mostly studied at the transcriptional level. Transcriptome studies in rice following *M. oryzae* infection have helped to identify the candidate genes involved in mediating resistance against pathogen^7^. Such studies also help to identify different sets of genes that regulate effector-triggered immunity (ETI) and pathogen associated molecular patterns (PAMPs) -triggered immunity (PTI). During ETI and PTI, complex networks of signalling pathway cascades are activated. The cascade involves respiratory bursts, activation of hormones, and kinase-mediated signalling, that activates various transcription factors (TF), which, in turn, activates pathogenesis-related (PR) genes. Activation of genes involved in defence leads to the synthesis of secondary metabolites and antimicrobial compounds like phytoalexins and phenolics etc. The defence-signalling pathways in both ETI and PTI can cross-talk and show some basic similarities^7–9^. Network modelling of genes playing important role in host-pathogen interaction helps to identify the regulatory network^10^. Only a few published studies deal with the transcriptome in rice upon *M. oryzae* infection^6^. Among them five studies have used rice NILs carrying single blast resistant genes to understand the mechanism mediated by the single gene upon *M. oryzae* infection^6,7^. Very high quality genome sequences of Nipponbare cultivar of *Oryza sativa* ssp. *japonica*^11^ and of *M. oryzae*^12^ are present in the public domain, which is regularly curated and updated. These attributes of both genomes make them particularly suitable for transcriptome profiling using RNAseq. Our group identified, mapped, and cloned a dominant blast-resistance gene, namely *Pi54*, which is resistant to several isolates of *M. oryzae*^13,14^ and then used marker assisted selection (MAS) to transfer *Pi54* along with two more blast-resistance genes, namely *Pi9* and *Pi1*, in Pusa basmati 1(PB1) background^15^. These resistant NILs have been evaluated across different regions of India^15^. Gene-linked marker AP5659-5 and functional marker NBS2-Pi9 195-1^16^ were used to confirm the presence of *Pi9* gene in PB1 + *Pi9* NIL while gene-linked markers, RM206^13^ and RM224^17^ were used to confirm the presence of *Pi54* and *Pi1* genes in PB1 + *Pi54* and PB1 + *Pi1* NILs, respectively. Background selection and foreground recombination were performed in PB1 + *Pi9*, PB1 + *Pi54*, and PB1 + *Pi1* to decrease linkage drag and to increase parental genome recovery, which reached 93.7% for *Pi9* and 92.7% for *Pi1* and *Pi54* in BC_3_F_1_ generations^15^. A transgenic line containing *Pi54* was used earlier for studying, with microarray technology, the defence mechanism mediated by *Pi54*^18^, and a resistant NIL of *Pi9* was studied, with microarray and RNAseq technologies^7,19^. The objective of present study was to compare the transcriptional level changes in PB1 and its three NILs carrying *Pi1*, *Pi9*, and *Pi54* genes upon *M. oryzae* infection using RNA-seq.

## Results

### Phenotyping of resistant NILs against different *M. oryzae* isolates

Seven NILs, namely PB1 + *Pi9*, PB1 + *Pi54*, PB1 + *Pi1*, PB1 + *Pita*, PB1 + *Pi5*, PB1 + *Pib*, and PB1 + *Piz5*, were phenotyped using six strains of *M. oryzae* (Supplementary Table 1). The lines resistant to different isolates of *M. oryzae* showed dark brown spots on the leaves. All the NILs were evaluated for their reaction to blast resistance using a 0–5 disease rating scale^20^, were 0 = resistant and 5 = susceptible, seven days after inoculation. The phenotyping reaction of different NILs like PB1 + *Pi9*, PB1 + *Pi54*, PB1 + *Pi1*, PB1 + *Pita*, PB1 + *Pi5*, PB1 + *Pib*, PB1 + *Piz5* was found to be 0,1,2,2,0,1,4, respectively against the *M. oryzae* strain Mo-nwi-53. The recurrent parent PB1 showed the characteristic spindle-shaped lesions with a disease rating of 5 and was considered as highly susceptible.

### Comparative genome-wide expression profiling

**Figure 1:**
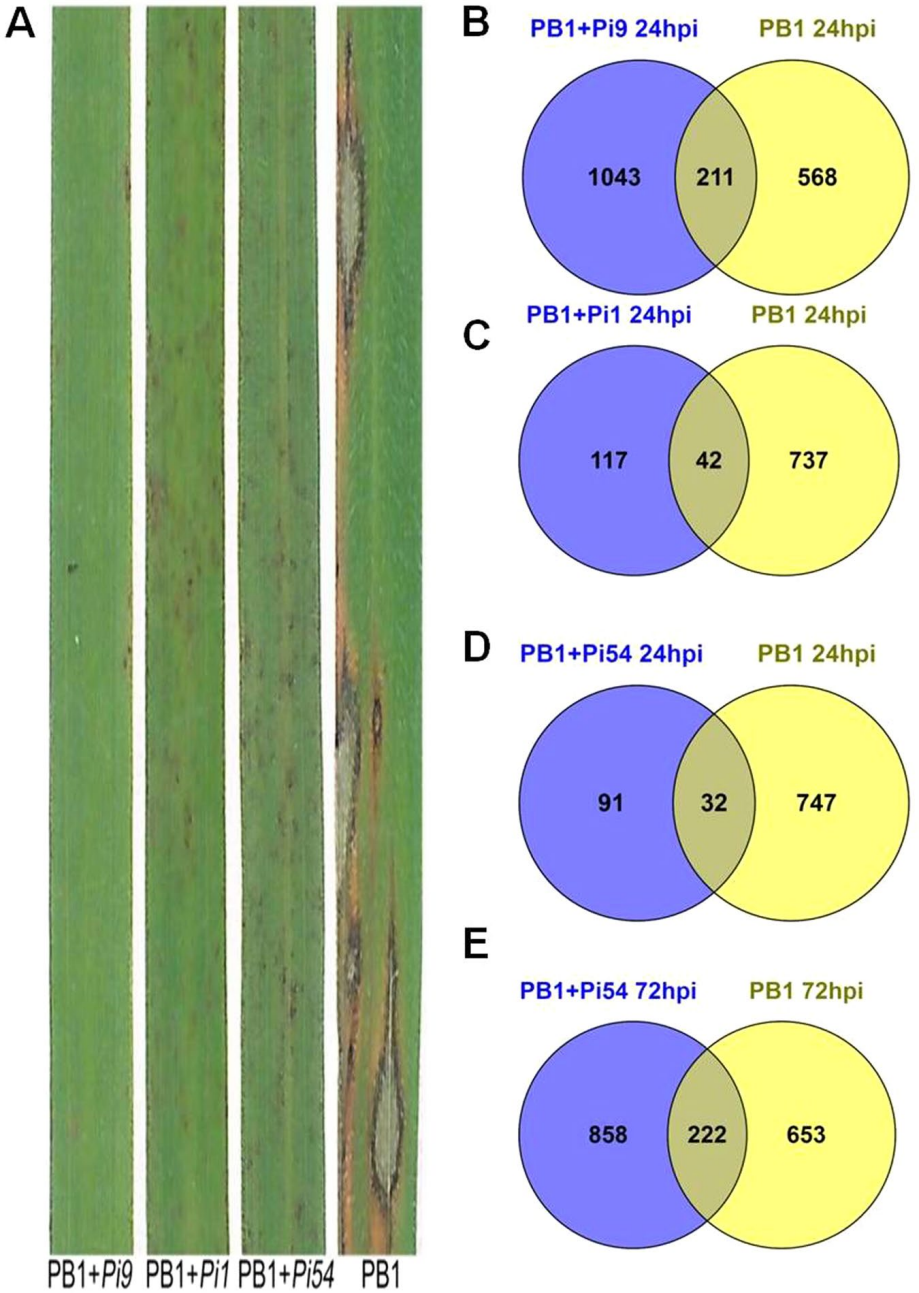
Expression profile of resistant NILs PB1 + *Pi9*, PB1 + *Pi1*, PB1 + *Pi54* and susceptible line PB1 with *Magnaporthae oryzae*. (**A**) Incompatible reaction of PB1 + *Pi9*, PB1 + *Pi1* and PB1 + *Pi54* NILs and compatible reaction of PB1. Comparison of significant (FDR adjusted *p* ≤ 0.05) differentially expressed loci (log_2_fold change ≥2) common between PB1 + *Pi9* 24 hpi and PB1 24 hpi (**B**), PB1 + *Pi1* 24 hpi *and* PB1 24 hpi (**C**), PB1 + *Pi54* 24 hpi and PB1 24 hpi (**D**), PB1 + *Pi54* 72 hpi and PB1 72 hpi (**E**).

**Figure 2:**
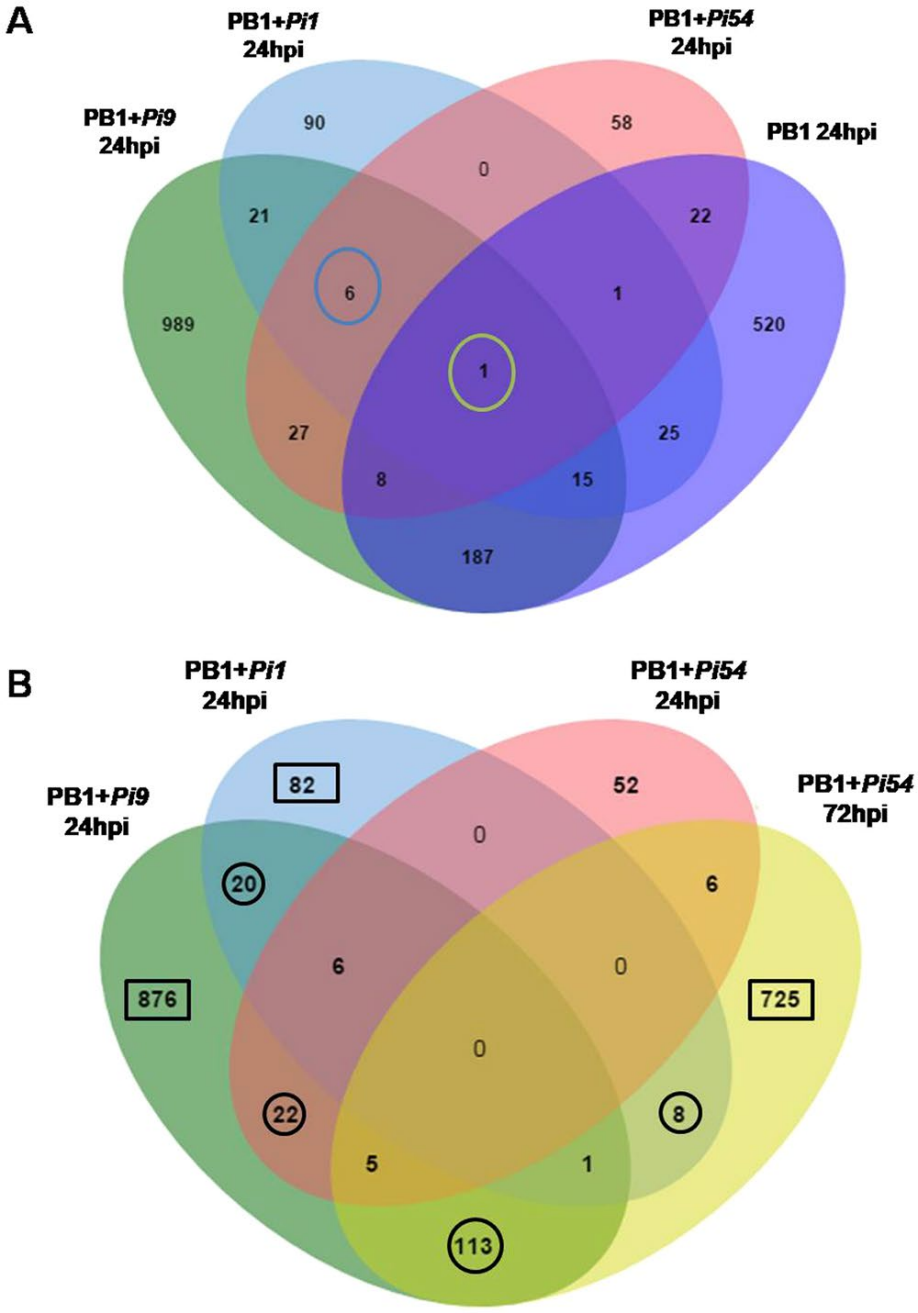
Comparative analysis of significant differentially expressed loci in different NILs. (**A**) Comparison of significant (FDR adjusted p-value ≤0.05) differentially expressed loci (log_2_fold change ≥2) between all three resistant NILs and susceptible control at 24 hpi. (**B**) Comparison of significant differentially expressed loci (SDEL) unique to each resistant NILs (compared to respective susceptible control) to show SDEL common (shown with circle) between NILs and SDEL specific (shown with rectangle) to each NIL.

**Figure 3:**
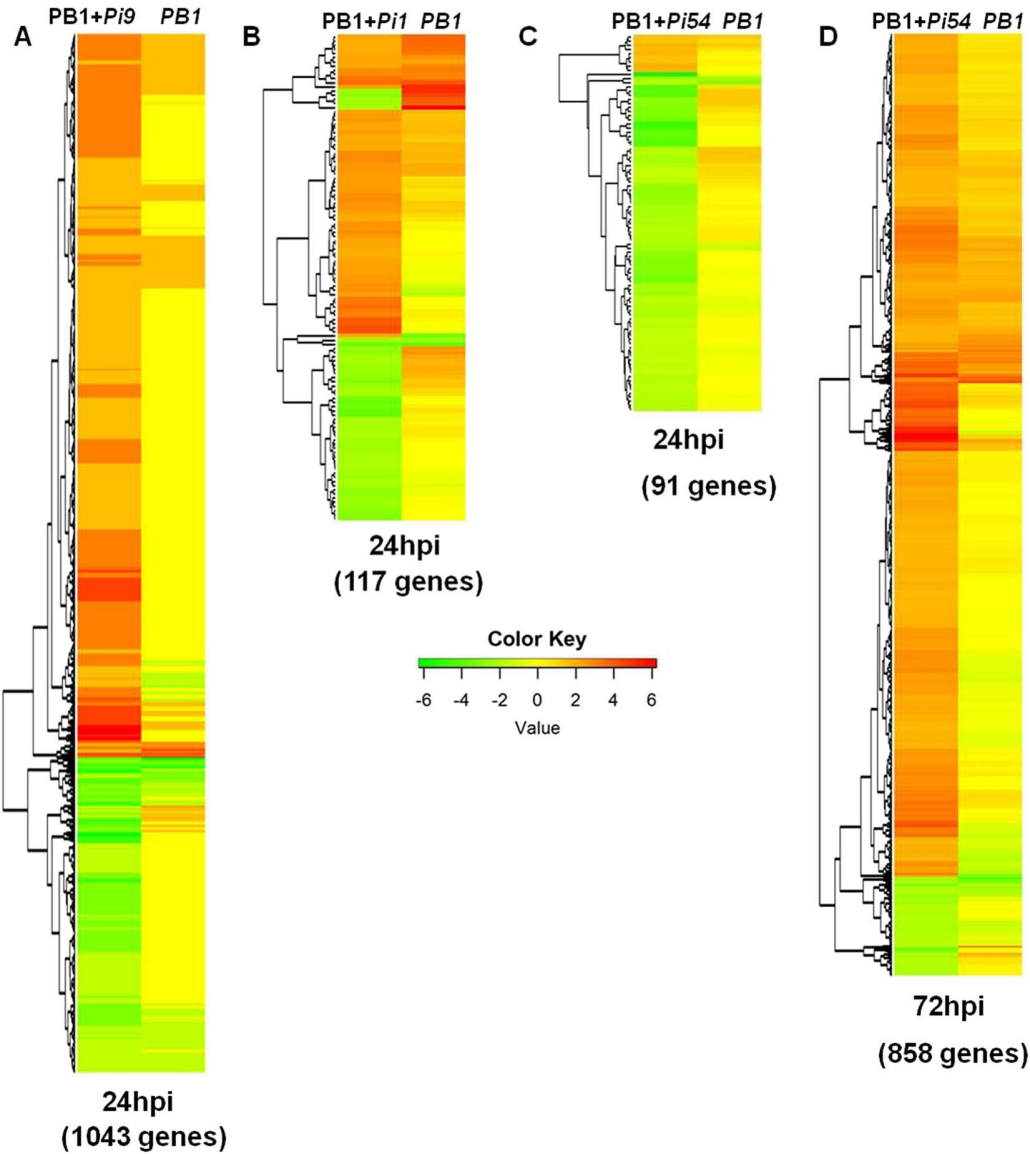
Heat map of significant differentially expressed unique loci of resistant NIL PB1 + *Pi9* 24 hpi and their respective log_2_fold change in PB1 24 hpi (**A**), PB1 + *Pi1* 24 hpi and their respective log_2_fold change in PB1 24 hpi (**B**), PB1 + *Pi54* 24 hpi and their respective log_2_fold change in PB1 24 hpi (**C**), PB1 + *Pi54* 72 hpi and their respective log_2_fold change in PB1 72 hpi (**D**). Red colour represent up-regulated loci and green colour represent down-regulated loci.

The three resistant NILs (PB1 + *Pi9*, PB1 + *Pi54*, and PB1 + *Pi1*) showed approximately 31 million pairs of filtered reads in each biological replicate (Supplementary Table 2). These pre-processed reads showed, on average, 88% alignment with the rice genome (version 7) available at Rice Genome Annotation Project^11^. All biological replicates (both treated and untreated samples) were correlated (Pearson’s R > 0.6), reflecting reproducibility. The number of significant differentially expressed loci (SDEL) in resistant NILs with a log_2_fold change ≥2 was 1254 (850 upregulated and 404 downregulated), 159 (90 upregulated and 69 downregulated), and 123 (22 upregulated and 101 downregulated) in PB1 + *Pi9*, PB1 + *Pi1*, and PB1 + *Pi54* 24 hours post inoculation (hpi), respectively, while in PB1 + *Pi54* 72 hpi number of SDEL were 1080 (975 upregulated and 106 downregulated). In the susceptible control PB1, SDEL with log_2_fold change ≥2 were 779 (466 up-regulated and 313 down-regulated) and 877 (560 upregulated and 317 downregulated) 24 hpi and 72 hpi, respectively. Large numbers of SDEL were found in the resistant NILs PB1 + *Pi9* 24 hpi and PB1 + *Pi54* 72 hpi and many were common to specific pairs (Fig. 1). Only one SDEL (LOC_Os08g08970) was shared by all three resistant NILs, along with susceptible PB1 at 24 hpi (Fig. 2A). Six SDEL with log_2_fold change ≥2 (LOC_Os02g45450, LOC_Os08g35110, LOC_Os04g48350, LOC_Os04g51460, LOC_Os02g52040, and LOC_Os06g48160) were found common between all three resistant NILs 24 hpi of *M. oryzae* (Fig. 2A). Among the six common loci, two were from glycosyl hydrolase family, two from dehydration responsive element binding proteins (DREB), one each from auxin-responsive gene family and phosphate induced gene (Supplementary Table 3). Among the six, three showed co-expression interactions in the network (Supplementary Table 4). The network showed enrichment of transcription factor activity (GO:0003700; FDR = 0.0436). The non-interacting loci were LOC_Os02g52040 (4330781), LOC_Os08g35110 (4345736), and LOC_Os06g48160 (4341940) (Supplementary Fig. 1). There was no common locus between PB1 + *Pi9*, PB1 + *Pi1*, PB1 + *Pi54* PB1 at 24 hpi; PB1 + *Pi54* and PB1 at 72 hpi. The number of SDEL unique to PB1 + *Pi9*; PB1 + *Pi1*; PB1 + *Pi54* at 24 hpi and PB1 + *Pi54* 72 hpi compared to susceptible control were 1043, 117, 91 and 858, respectively, (Figs 1B, 3 & Supplementary Table 5).

### Functional annotation analysis of SDEL unique to each resistant NILs

**Figure 4:**
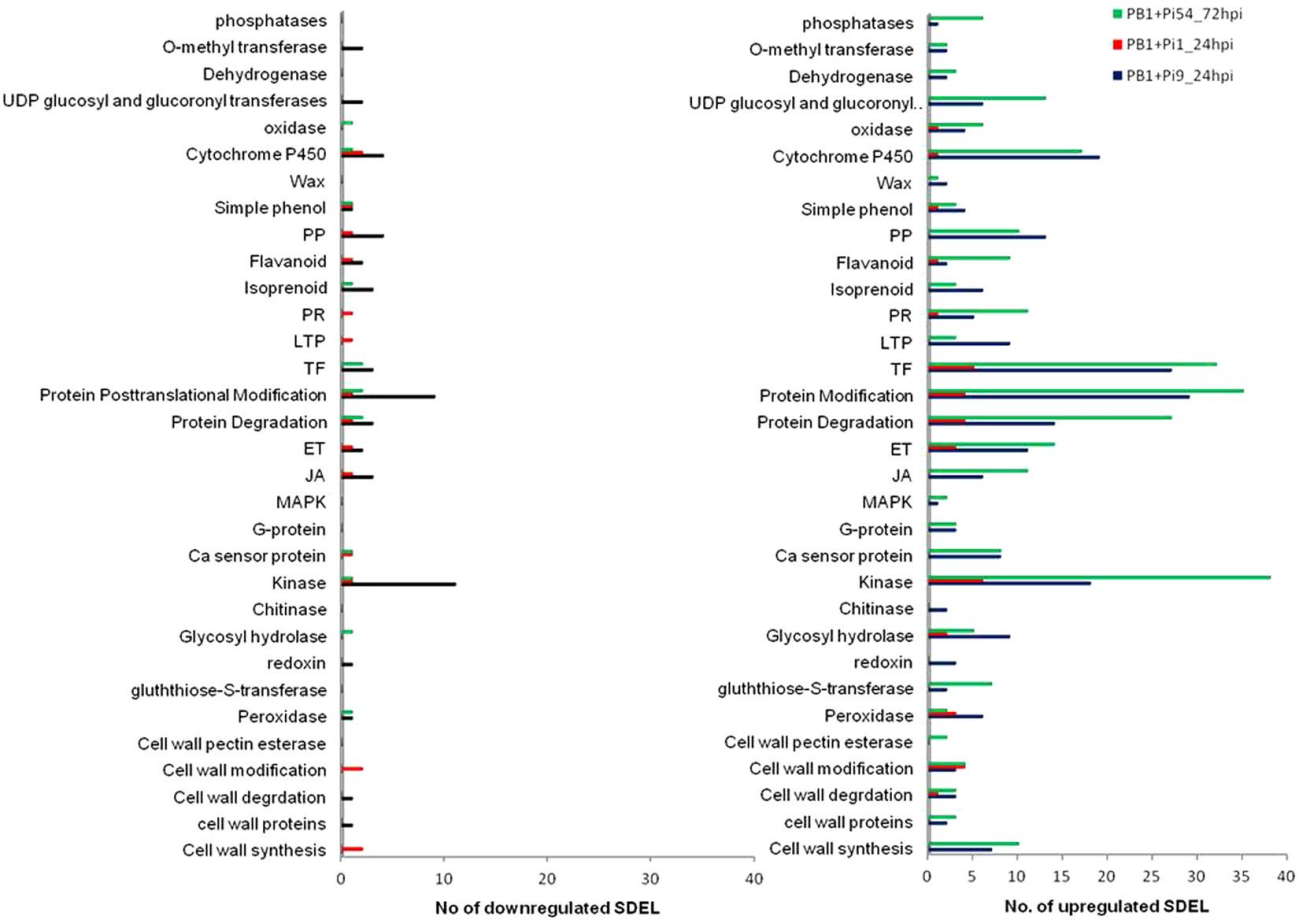
Number of up and down regulated unique SDEL of resistant NILs PB1+*Pi9* 24 hpi; PB1 + *Pi1 24* hpi and *PB1* + *Pi54* 72 hpi falling in important gene categories induced in rice upon *M. oryzae* infection.

Functional annotation of SDEL unique to the resistant NILs PB1 + *Pi9*, PB1 + *Pi1* both at 24 hpi and PB1 + *Pi54* after 72 hpi showed that the number of upregulated SDEL was much greater than that of downregulated SDEL in different functional categories (Fig. 4). Higher number of genes related to the synthesis and the degradation of the cell wall were upregulated in the resistant NILs PB1 + *Pi9* and PB1 + *Pi54* compared to PB1 + *Pi1*; cell wall pectin genes were upregulated only in PB1 + *Pi54*; and cell wall modification genes were upregulated in all three resistant NILs. Similarly, genes related to respiratory burst, such as peroxidase that scavenge reactive oxygen species (ROS) were upregulated in all three resistant NILs whereas glutathione S-transferase was upregulated only in PB1 + *Pi9* and PB1 + *Pi54*. Beta 1,3 glucanase proteins, which degrades fungal cell walls by hydrolytic activity was upregulated in all three resistant NILs, as were the genes related to signalling mediated by kinase and hormones. The number of kinase genes upregulated in PB1 + *Pi54* was double of that in PB1 + *Pi9*. G proteins, calcium sensor protein and mitogen activated protein kinases (MAPK), which are involved in signalling, were upregulated in PB1 + *Pi9* and PB1 + *Pi54* but absent in PB1 + *Pi1*. In the case of hormones, the genes related to both jasmonic acid (JA) and ethylene (ET) signalling were upregulated in PB1 + *Pi9* and PB1 + *Pi54* whereas in PB1 + *Pi1*, only those related to ET signalling were upregulated and those related to JA signalling were absent. Genes related to protein degradation and modification was found to be upregulated in higher number in PB1 + *Pi9* and PB1 + *Pi54* compared to PB1 + *Pi1*. Transcription factors activated upon kinase and hormone signalling were also upregulated in greater numbers in PB1 + *Pi9* and PB1 + *Pi54* compared to PB1 + *Pi1*. The number of transcripts activated in genes related to PR, such as LTP and chitinase, were greater in PB1 + *Pi9* and PB1 + *Pi54* than in PB1 + *Pi1*. Different classes of enzymes like phosphatases, o-methyl transferase, dehydrogenase, Uridine 5′-diphospho (UDP) glucoronosyl and UDP-glucosyl transferase were upregulated in PB1 + *Pi9* and PB1 + *Pi54* whereas oxidase was upregulated in all three resistant NILs. Cytochrome P450 loci were upregulated and present in much greater numbers in PB1 + *Pi9* and PB1 + *Pi54* than in PB1 + *Pi1*. Lastly, secondary metabolites such as phenylpropanoid, flavonoid, isoprenoid and simple phenol were highly upregulated mainly in PB1 + *Pi9* and PB1 + *Pi54*.

### Gene ontology enrichment analysis revealed biological processes associated with defence response to *M. oryzae* in the resistant NILs

**Figure 5:**
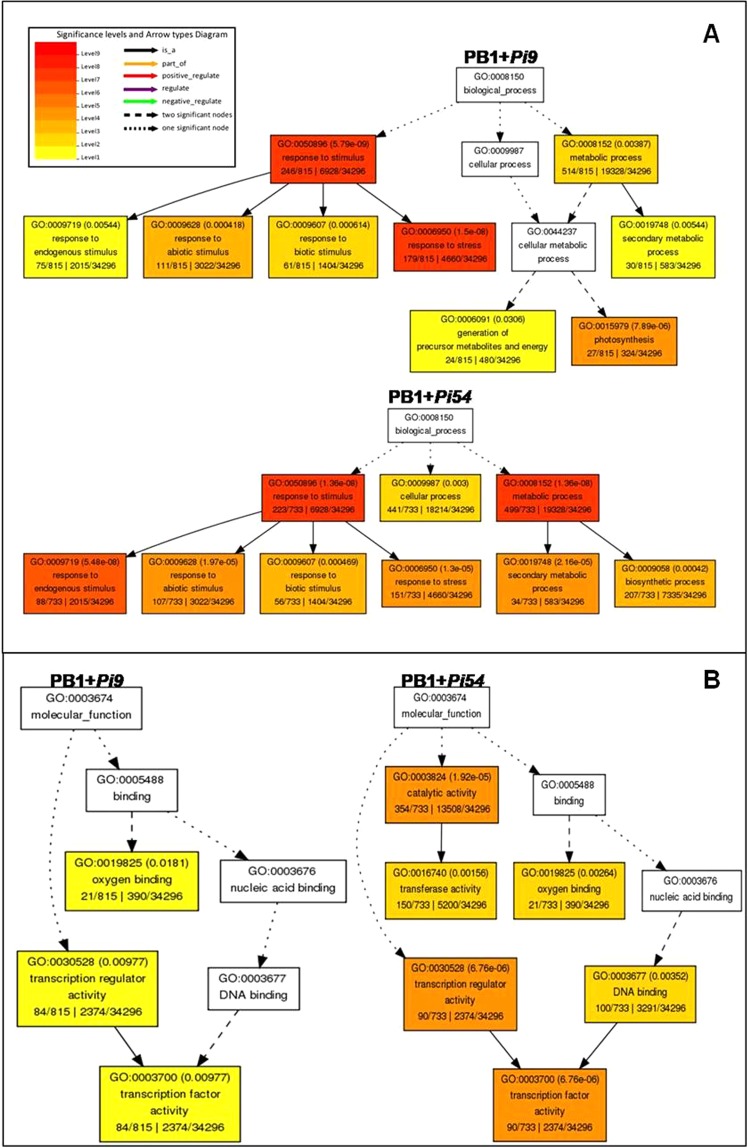
Geneset enrichment analysis of significant biological processes (**A**) and molecular functions (**B**) enriched in differentially expressed loci unique to NILs PB1 + *Pi9* 24 hpi and PB1 + *Pi54* 72 hpi.

The data on singular enrichment analysis (SEA) of unique SDEL in resistant NILs, generated GO hieratical graph containing statistically significant GO terms (Fig. 5). The biological processes enriched using SEA in PB1 + *Pi9* 24 hpi were response to stimulus (GO:0050896) and metabolic processes (GO:0008152). The response to stimulus included response to endogenous stimulus (GO:0009719), response to biotic stimulus (GO:0009607) and response to other stresses (GO:0006950). The metabolic process included secondary metabolic process (GO:0019748) and cellular metabolic process that in turn included generation of precursor metabolites (GO:0006091) and photosynthesis (GO:0015979). In PB1 + *Pi54* 72 hpi, the biological process enriched were similar to PB1 + *Pi9* except GO:0006091 and GO:0015979 (Fig. 5 & Supplementary Table 6). The enriched molecular function using SEA in PB1 + *Pi9* 24 hpi were transcription regulator activity (GO:0030528), oxygen binding (GO:0019825) and transcription factor activity (GO:0003700). Besides, three additional molecular functions like DNA binding (GO:0003677), catalytic (GO:0003824) and transferase activity (GO:0016740) were also enriched in PB1 + *Pi54* 72 hpi (Fig. 5 & Supplementary Table 7).

### Comparison of unique SDEL among resistant NILs

Among the SDEL unique to each resistant NIL (as compared to the control), 20 were common between PB1 + *Pi9* 24 hpi and PB1 + *Pi1* 24 hpi; 22, between PB1 + *Pi9* 24 hpi and PB1 + *Pi54* 24 hpi; 8, between PB1 + *Pi1* 24 hpi and PB1 + *Pi54* 72 hpi and 113 were common between PB1 + *Pi9* 24 hpi and PB1 + *Pi54* 72 hpi (Fig. 2B). Among the 113 SDEL, two (LOC_Os05g07870.1 and LOC_Os05g47770) showed novel alternate splice variants.

**Figure 6:**
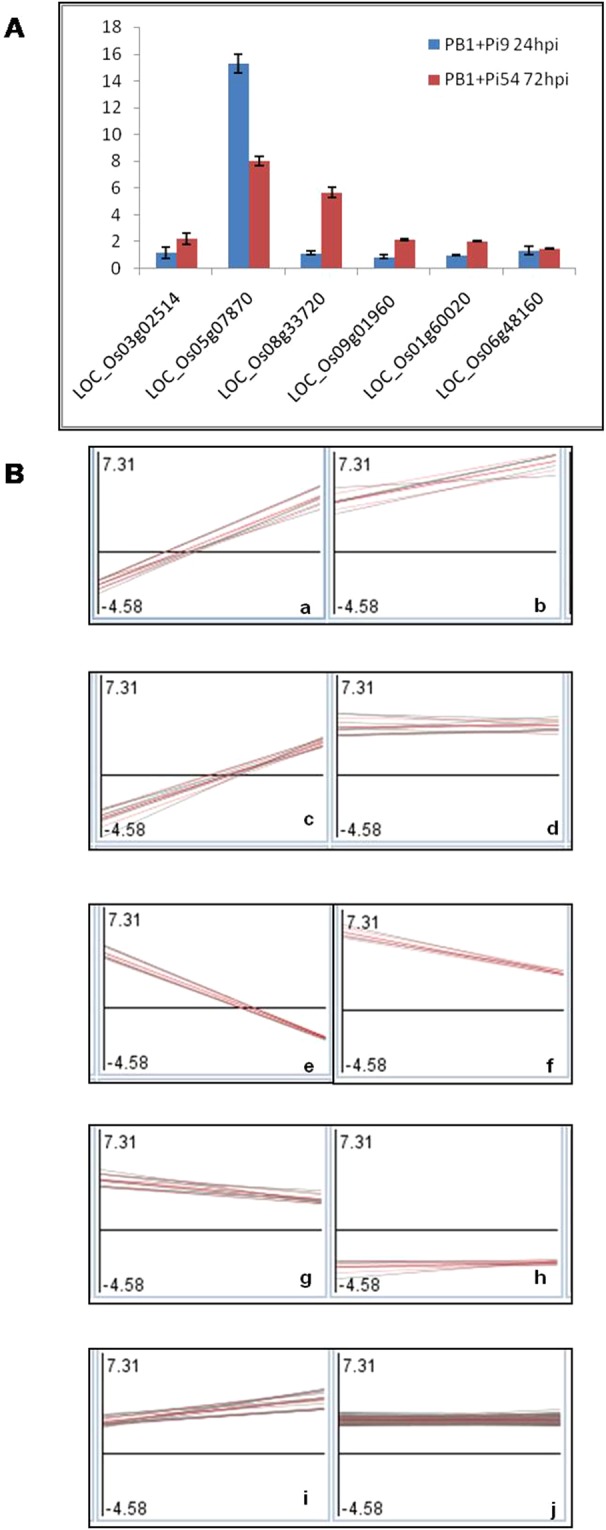
Validation and cluster analysis of SDEL. (**A**) The qRT-PCR validation of significant differentially expressed loci (SDEL) in PB1+*Pi9* 24 hpi and PB1 + *Pi54* 72 hpi of *M. oryzae* infection. (**B**) Cluster analysis of SDEL common between resistant NILs PB1 + *Pi9* 24 hpi and PB1+*Pi54* 72 hpi, to study the similar expression pattern between them.

All the 113 common SDELs were involved in primary and secondary metabolism and hormone synthesis. The primary metabolism included synthesis and degradation of starch, synthesis and elongation of fatty acids, and hydrolysis of phospholipids. Whereas secondary metabolism included the synthesis of phenylpropanoid; and hormone synthesis included the synthesis of ethylene and JA (Supplementary Table 8). In both PB1 + *Pi9* 24 hpi and PB1 + *Pi54* 72 hpi, more than 2 fold higher expression was obtained for the SDEL related to starch synthase, glycosyl hydrolase, 3-ketoacyl-CoA synthase and S-adenosyl methionine synthetase involved in starch synthesis; starch degradation; synthesis and elongation of fatty acid; ethylene synthesis respectively. Similarly upregulation of two loci of 12-oxophytodienoate reductase (involved in JA synthesis), one locus of caffeoyl-CoA O-methyl transferase (involved in phenylpropanoid synthesis), was also obtained in these two NILs after 24 and 72 hpi (Supplementary Table 8). The expression of few common SDEL was validated using qRT-PCR in PB1 + *Pi9* 24 hpi and PB1 + *Pi54* 72 hpi of *M. oryzae* and the results are depicted in Fig. 6A & Supplementary Table 9. The SDEL common between PB1 + *Pi9* and PB1 + *Pi54* were clustered in ten sets of clusters (Fig. 6B & Supplementary Table 10). Among them, six clusters showed similar trends in upregulation; one cluster showed similar trends in downregulation; and three clusters showed opposite trends of regulation. Eight small clusters (cluster 1 to 8) were obtained which contained loci involved in primary metabolism (starch and lipid), transcription factors, and PR proteins. The large clusters (clusters 9 and 10) contained signalling loci such as kinases, hormones and G- proteins, loci involved in primary (amino acid) metabolism, secondary metabolism, hormone metabolism, signalling kinases, transcription factors and protein degradation.

### Network analysis of SDEL common between PB1 + *Pi9* 24 hpi and PB1 + *Pi54* 72 hpi

**Figure 7:**
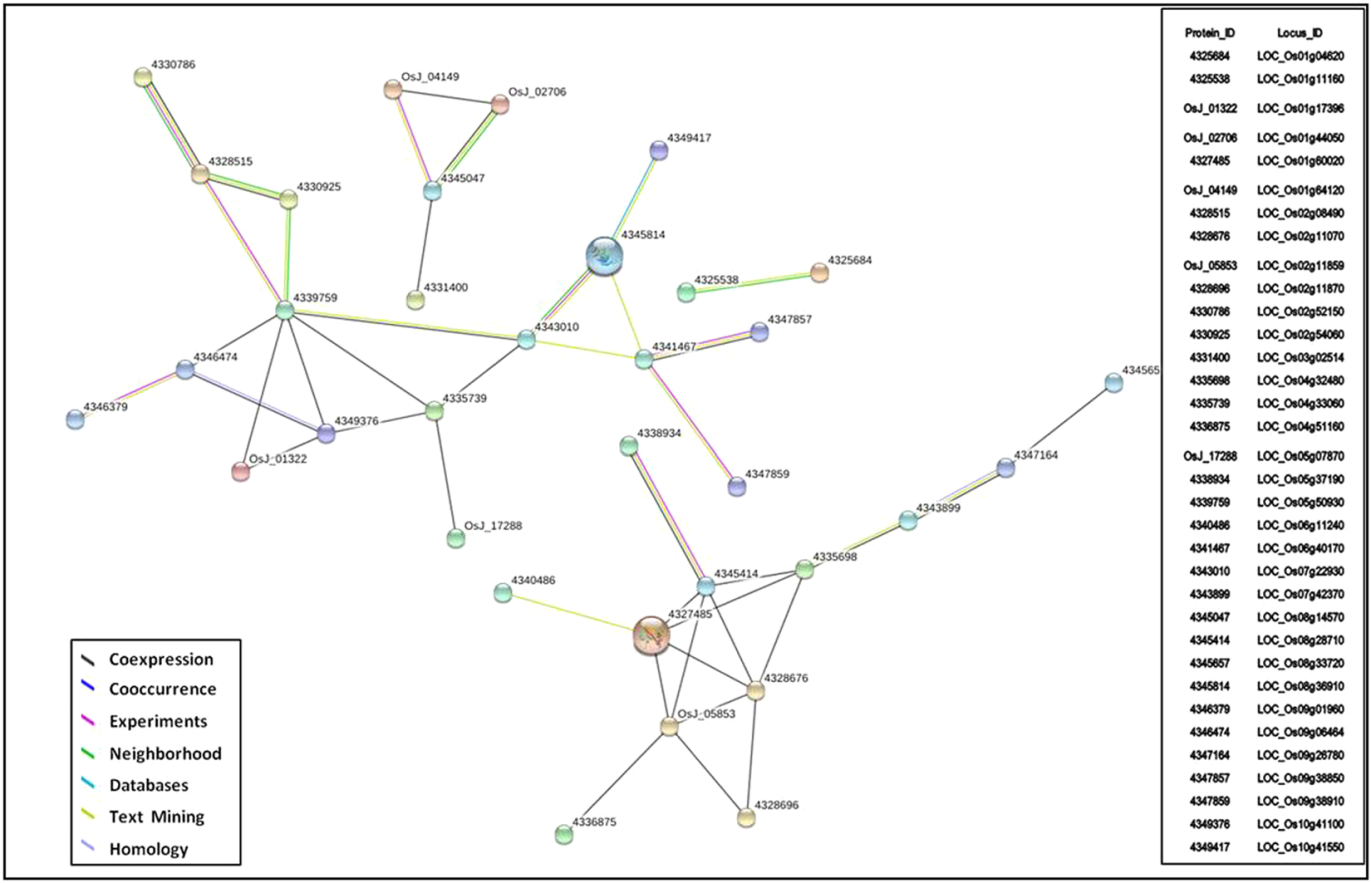
Co-expression network of proteins corresponding to unique significant loci common between resistant NILs PB1 + *Pi9* 24 hpi and PB1 + *Pi54* 72 hpi.

Among the proteins encoded by 113 SDEL, 34 proteins showed 43 predicted associations in the network (Fig. 7 and Supplementary Table 11); 32 showed co-expression associations; 8 were validated experimentally and 19 showed text-mining associations. The combined score was 0.4 for 9 associations in the network; the remaining 34 had combined score above 0.4. The 34 proteins presented in the network mainly included zinc finger protein, hydrolase, NAD dependent epimerase, triose phosphate, phospholipase D, dehydrogenase, MYB, and OsWAK. The three zinc finger proteins present in the network were co-expressed and were involved in the hormone signal transduction pathway of the plants (Supplementary Table 12).

### Pathway analysis of SDEL specific to each resistant NILs

Each of the three resistant NILs had SDEL that were specific to itself, not shared with any other NIL: PB1 + *Pi9* had 876 such SDEL, PB1 + *Pi1* had 82, and PB1 + *Pi54* had 725 (Fig. 2B).

Annotation of SDEL specific to each NIL reveal that overall pathway involved in biotic stresses were highly up and downregulated in the NILs containing *Pi9* and *Pi54* genes compared to the NIL with *Pi1*. These biotic stress related pathways mainly included loci related to activation of respiratory burst, cell wall related enzymes, signaling molecule like hormones or kinases, transcription factors and defense-related genes. Among the loci related to respiratory burst, in PB1 + *Pi1* only peroxidases showed differential expression whereas in PB1 + *Pi9* and PB1 + *Pi54*, change in expression level was observed in peroxidase, thioredoxin, glutathioredoxin, and glutathione-S transferase (Supplementary Fig. 2).

SDEL related to the synthesis as well as the degradation of the cell wall, and those related to cell wall proteins, were differentially expressed in all three NILs, but their numbers were much higher in PB1+*Pi9* and PB1 + *Pi54* than in PB1 + *Pi1*. Beta glucanase showed differential expression in PB1 + *Pi9* and PB1 + *Pi54* but not in PB1 + *Pi1*.

With respect to signalling hormones, only two SDEL related to ET metabolism were differentially expressed in PB1+*Pi1* whereas in PB1 + *Pi9* and PB1 + *Pi54*, several SDEL related to both ET and JA metabolism were differentially expressed. Signalling kinases showed changes in expression in all three NILs. Few transcription factors were differentially expressed in PB1 + *Pi1* but several (included WRKY, MYB, and ERF) were differentially expressed in PB1 + *Pi9* and PB1 + *Pi54*. In PB1 + *Pi9* and PB1 + *Pi54*, several SDEL related to both primary metabolism (photosynthesis and the metabolism of carbohydrates, lipids, and proteins) and secondary metabolism (phenylpropanoids, lignins, phenolics, flavanoids, waxes, and terpenes) showed significant differential expression while very few in PB1 + *Pi1* (Supplementary Fig. 3).

**Figure 8:**
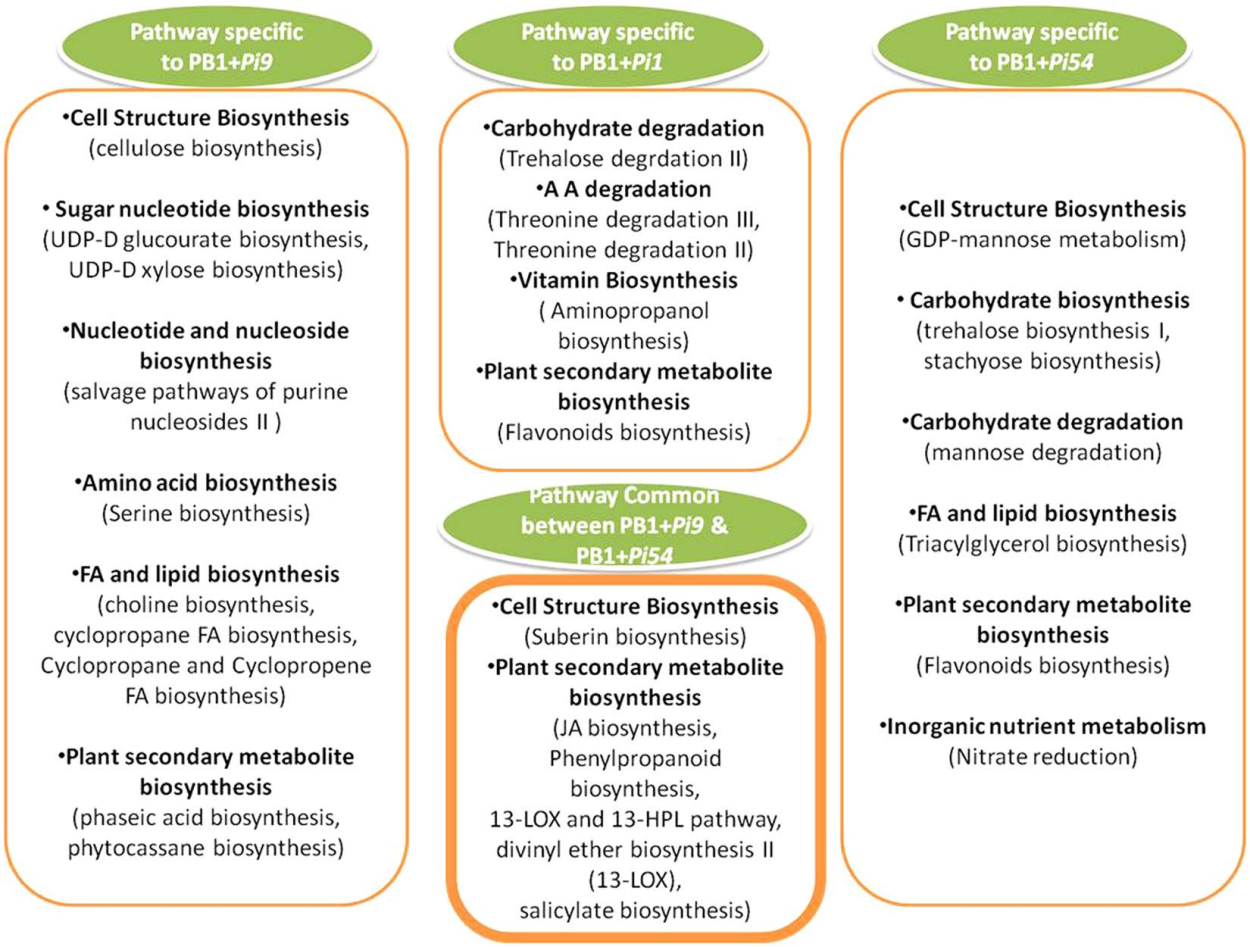
Significantly (p-value <0.05) enriched pathway specific to resistant NILs, PB1 + *Pi9* 24 hpi; PB1 + *Pi1 24* hpi and PB1 + *Pi54* 72 hpi, respectively.

A detailed metabolic pathway analysis of the 82 SDEL specific to PB1 + *Pi1* showed significant changes in trehalose degradation II, threonine degradation III, threonine degradation II, aminopropanol biosynthesis and flavonoids biosynthesis. Similarly, the analysis of the 876 SDEL specific to PB1 + *Pi9* showed significant changes in the pathways related to cellulose biosynthesis, UDP-D glucourate biosynthesis, UDP-D xylose biosynthesis, salvage pathways of purine nucleosides II, serine biosynthesis, choline biosynthesis, cyclopropane FA biosynthesis, cyclopropane and cyclopropene FA biosynthesis, phaseic acid biosynthesis, and phytocassane biosynthesis. Pathway analysis of 725 SDEL specific to PB1 + *Pi54* NILs 72 hpi showed significant changes in GDP-mannose metabolism, trehalose biosynthesis I, stachyose biosynthesis, mannose degradation, triacylglycerol biosynthesis, flavonoids biosynthesis, and nitrate reduction pathways. The JA biosynthesis, salicylate biosynthesis, 13-lox and 13-hpl, divinyl ether biosynthesis II, phenylpropanoid biosynthesis, and suberin biosynthesis pathways were common between PB1 + *Pi9* 24 hpi and PB1 + *Pi54* 72 hpi (Fig. 8 and Supplementary Fig. 4).

## Discussion

Phenotyping of seven rice blast resistant NILs, namely PB1 + *Pi9*, PB1 + *Pi54*, PB1 + *Pi1*, PB1 + *Pita*, PB1+*Pi5*, PB1 + *Pib*, and PB1 + *Piz5* using six strains of *M. oryzae* helped in understanding the pattern of virulence of the strains and the spectrum of resistance in the NILs. Phenotyping of PB1 + *Pi9*, PB1 + *Pi1*, and PB1 + *Pi54* showed that these three NILs were more suitable for studying the response of rice resistance genes to *M. oryzae* infection. The constitutively expressed genes *Pi9* and *Pi1* are not induced upon pathogen infection^16,21^. The constitutively expressed *R* gene, upon infection by *M. oryzae*, remodels post-transcriptional changes that activate downstream processes involved in coping with biotic stresses. The pathogen inducible *Pi54* gene is expressed constitutively at a basal level up to 48 hpi and gets induced by *M. oryzae* infection at 72 hpi in resistant line^10^. We therefore undertook transcriptome profiling of all three blast resistant NILs (PB1 + *Pi9*, PB1 + *Pi54*, PB1+*Pi1*) 24 hpi. In addition expression profiling of PB1 + *Pi54* 72 hpi was also performed. Comparative transcriptome profiling of all three resistant NILs shows that number of unique SDEL are much higher in PB1 + *Pi9* (24 hpi) and PB1 + *Pi54* (72 hpi) compared to PB1 + *Pi1* (24 hpi). This suggests that different sets of genes for resistance are induced when a specific blast-resistance gene is introduced in the rice line with a common genetic background (PB1). The fact that only one locus was common between the resistant and the susceptible lines show that different sets of genes mediate the reactions that govern resistance and susceptibility. Six unique (compared to control) SDEL were common to all three NILs because these represent a set of common loci that are induced in rice upon infection by *M. oryzae* at 24 hpi. Among them the proteins encoded by three SDEL were co-expressed with five other rice proteins present in the rice network. The three co-expressing proteins showed enrichment of transcription factor activity a reflection of their importance in controlling the regulation of downstream genes governing the defensive response of the plant. This interactive network has three important candidate proteins that are commonly induced in resistant NILs 24 hpi with *M. oryzae*.

In all three resistant NILs, functional annotation of unique SDEL helped to identify the genes related to different cellular processes that are activated upon infection by *M. oryzae*. These processes include depolarization of the plasma membrane, generation of ROS and antimicrobial compounds, activation of MAPK, deposition of lignin and callose along the cell wall as well as the transcription of PR genes. The functional annotation of SDEL helped to understand the role of all these loci in different metabolic pathways during defense response against *M. oryzae*.

Singular enrichment analysis of SDEL unique to each resistant NIL showed that the number of molecular functions and biological processes enriched in PB1 + *Pi9* and PB1 + *Pi54* was greater than that in PB1 + *Pi1*. A total of ten molecular functions and biological processes were common between PB1 + *Pi9* and PB1 + *Pi54*. This analysis supports functional annotation and shows that activation of transcriptional regulatory activity is crucial to the transcription of important PR proteins and secondary metabolites involved during biotic stress response in resistant NILs upon fungal inoculation compared to the susceptible control. The comparison of SDEL unique to each NIL (compared to the control) showed that the number of common SDEL (113) was the highest between PB1 + *Pi9* and PB1 + *Pi54*. The clustering of those SDEL reflects the similarities between the two NILs in terms of the regulation of key molecules involved in signalling, primary and secondary metabolism, transcription factors, and protein degradation.

In network analysis of SDEL common between PB1 + *Pi9* 24 hpi and PB1 + *Pi54* 72 hpi, nine associations, with a score of 0.4, showed medium confidence whereas 34 associations, with scores above 0.4, showed high confidence. Co-expression, text mining, and experimental association with a high confidence level in the network mark important candidate genes that are induced in resistant NILs upon infection with *M. oryzae*. Among the important candidates, the role of zinc finger proteins is particularly prominent because all of them are co-expressed and involved in the hormone signal transduction pathway.

Among the 113 SDEL common to PB1 + *Pi9* 24 hpi and PB1 + *Pi54* 72 hpi, a novel alternative splice variant of LOC_Os05g47770, which is a serine protein kinase (At1g18390) precursor, changed its expression upon *M. oryzae* inoculation in both the NILs. The LRK10 gene present at locus At1g18390 in *Arabidopsis* is closely related to Wheat Rust 10 disease resistance locus like protein kinase. The AtLRK10L1.2 transcript had several splice variants^22^. The transcript LOC_Os05g47770, which showed changes in the expression of the splice variant, probably acts in ways similar to those seen in the *Arabidopsis* leaf rust receptor like kinase. Detailed pathway analysis of SDEL specific to each NIL show common pathways between PB1 + *Pi9* 24 hpi and PB1 + *Pi54* 72 hpi. Each NIL have several specific SDEL that are regulating different pathway. So pyramiding of *Pi9* and *Pi54* blast resistance gene in commercial varieties of rice can provide broad spectrum blast resistance.

Functional annotation of common SDEL falling in one cluster shows that these SDEL commonly code for primary as well as secondary metabolic pathways proteins, transcription factors, and genes related to signalling and protein degradation. The metabolic pathways, clustering, and co-expression network analysis present a clear picture of the defence mechanism in both the resistant NILs containing *Pi9* and *Pi54* genes. The gene clusters helped to find the common hubs of metabolic pathways that are part of the broad-spectrum resistance. The co-expression network of the proteins encoded by these common SDEL identified the actual set of candidate genes that together form the module. In a co-expression network, a set of genes interact within a module and also have discrete functions^23^. The network identified the actual module of interacting genes present in both the NILs to provide broad-spectrum resistance against *M. oryzae*. The broad-spectrum resistance mechanism, commonly observed in both the NILs might be due to the presence of blast resistance genes *Pi9* and *Pi54* in the genetic background of PB1. The correlation between transcript abundance as measured by microarray and proteome data is reported to be low (R = 0.24)^24^. Manipulation of targeted genes related to a specific trait will not generate a phenotype with broad-spectrum resistance. Metabolic pathway and proteomic analysis should be combined to accurately identify the candidate genes involved in plant response to biotic stresses^25,26^. If priority for genes in a breeding programme is to be assigned precisely, we need to find the relevant genes, assess their involvement in metabolic pathways in detail, and examine how they interact. This approach shows a potential path to crop engineering based on selected genes that are more likely to affect the trait of interest on a global scale. The present study showed overlapping as well as unique set of genes that are regulated in rice NILs providing broad spectrum resistance upon *M. oryzae* infection. The overlapping sets of a gene module or hub identified the actual signalling network that is activated following an attack by *M. oryzae* in different NILs none of which had a common rice blast resistant *R* gene. The molecular mechanisms of *Pi9, Pi54* and *Pi1* genes in a common genetic background will help in developing blast resistant rice varieties. A rice variety endowed by pyramiding with these blast resistance genes will be an agronomically important rice variety with high yield and will thus contribute to global food security.

## Methods

### Plant material and growth conditions

Three NILs of Pusa Basmati 1 (PB1; *O. sativa* ssp. *indica*), each with one gene for blast resistance, namely *Pi9*, *Pi1*, or *Pi54*, were used in the present study. These NILs were resistant to several isolates of *M. oryzae* whereas PB1 served as the susceptible control. We used seeds of the BC3F2 generation for PB1 +  *Pi1* and PB1 +  *Pi54* and those of the BC3F6 generation for PB1 + *Pi9*. The seeds of all three NILs and of the susceptible control were surface-sterilized and then germinated on moist filter paper for 5 days at 37 °C. The potting mixture consisted of soil mixed with vermicompost and gypsum in the ratio of 6:2:1 was autoclaved and used for growing the rice seedlings. The seedlings were grown in pots filled with autoclaved soil under standard growth conditions, at 25 ± 2 °C at 16 h of light (115 μmol m^−2^ s^−1^) alternating with 8 h of darkness. Two-week-old healthy plants (at the four-leaf stage) were inoculated with a highly virulent isolate (Mo-nwi-53) of *M. oryzae*.

### Fungal inoculation on rice lines

Cultures of *M. oryzae* strain Mo-nwi-53 which was initially collected from north-western India were maintained on potato dextrose agar (PDA), oatmeal agar, and Mathur’s media for 15 days (at 25 ± 1 °C). Conidia of *M. oryzae* were collected from the culture plates after rinsing with 0.25% gelatin. After filtering the conidial suspension through two layers of gauze, the concentration was adjusted to 10^5^ spores mL^−1^ (ascertained by using a haemocytometer). All the untreated plants of NILs and the susceptible control were fine sprayed with 0.25% gelatin while the treated plants were fine sprayed with *M. oryzae* suspended in 0.25% gelatin. The experiment comprised of three biological and three technical replicates each of both treated (inoculated) and untreated plants of all the three resistant NILs and the susceptible control line at four-leaf stage. The plants after inoculation were placed for 24 hours in dark in a humid chamber (90% relative humidity) at 25 ± 1 °C. Tissue samples were collected from the fully expanded leaves from both treated and untreated plants 24 hpi and immediately frozen in liquid nitrogen. Leaf tissue samples of PB1 + *Pi54* and the susceptible control were also collected at 72 hpi and frozen in liquid nitrogen.

A few leaves from each treatment and from the control were monitored for disease development and scored on a 0–5 disease rating scale; where 0 = resistant and 5 = susceptible^20^. Leaves of the susceptible control (PB1) had a disease rating of 5 after seven days of inoculation. The characteristic spindle-shaped lesions of rice blast were observed in the susceptible plants (Fig. 1A).

### RNA isolation and RNAseq library preparation

Total RNA was extracted from leaf tissues using Spectrum™ Plant Total RNA Kit (Sigma-Aldrich). The quantity of RNA was measured using a spectrophotometer (NanoDrop 1000, Thermo Fischer Scientific) and the quality of RNA was assessed through electrophoresis, using an Agilent 2100 Bioanalyzer (Agilent Technologies) and spectrophotometer(NanoDrop 1000). Samples of RNA with RIN (RNA Integrity Number) >8.5 were used for preparing the DNA library.

Total RNA samples (5 µg each) from each biological replicate of the resistant lines and the susceptible line were used for library preparation using TruSeq RNA Library Prep Kit ver. 2 (Illumina, San Diego, California, USA). The size and the quality of the library were assessed using Agilent 2100 Bioanalyzer and a high-sensitivity DNA kit. A paired-end sequencing run was performed on Illumina HiSeq 1000 using TruSeq SBS Kit ver. 3-HS (Illumina). A software package, namely CASAVA 1.7, was used for Bcl conversion and de-multiplexing. Low-quality reads and adapters were removed using Trimmomatics^27^ whereas read-quality were accessed using FastQC. The transcriptome data were deposited with NCBI’s Gene Expression Omnibus^28^ and are accessible through the GEO Series (Accession no. GSE117030).

### Bioinformatics analysis

An index of *O. sativa* ssp. *japonica* (the reference genome) was built using bowtie2-build module. In all three NILs and the susceptible control (PB1), a spliced-read mapper Tophat ver. 2.0.9^29^ that defines the exon–intron junctions was used for mapping the reads against *O. sativa* ssp. *japonica*^23^ (release 7). The aligned reads obtained from Tophat were analysed in Qualimap^30^ and visualized on an integrated genomic viewer (IGV)^31^. The reads were assembled into transcripts with reference annotations to guide the assembly using Cufflink version 2.1.1^32^. The following parameters were used during the assembly: average fragment length of 200, abundance estimation, fragment length standard deviation of 80, and unlimited alignment per fragment. For quantifying the SDEL, Cuffdiff^32^ (version 2.1.1) was used and analysed the output in CummeRbund. In Cuffdiff, transcripts are quantified in terms of fragments per kilobase of transcript per million mapped reads (FPKM). The transcripts identified from all expressed loci after applying multiple corrections (FDR adjusted *p* value ≤0.05) with log_2_fold change ≥2 were considered as the SDEL. Cuffdiff output was used to identify alternative splice variant. The dispersion model of the transcript was constructed using the pooled dispersion method, and those SDEL were identified that were uniquely expressed in the respective resistant NILs (PB1 + *Pi9*, PB1 + *Pi1*, PB1 + *Pi54*) but were absent in the susceptible control PB1 with log_2_fold change ≥2. The unique SDEL of all the NILs were compared to identify SDEL specific to each resistant NIL. Heatmap2 (a part of the software package *R*) was used for generating heat maps of unique SDEL in the resistant NILs. Venn diagrams comparing the resistant NILs and the susceptible control were generated in InteractiVenn (http://www.interactivenn.net/) and jvenn (http://jvenn.toulouse.inra.fr/app/index.html). Pearson correlation coefficients between biological replicates were calculated in *R*.

### Metabolic pathway and analysis of gene ontology

MapMan ver. 3.1.1^33^ was used for pathway analysis of SDEL unique to each resistant NIL as compared to the susceptible control and that of SDEL specific to each resistant NIL as compared to other resistant NILs. During the pathway analysis, classification and functional annotation of the SDEL was done against *Oryza sativa* (TIGR7 database). Singular enrichment analysis was performed on SDEL unique to the resistant NILs using AgriGO. Significant biological processes and molecular functions that fell into different GO annotations were identified among the uniquely expressed SDEL of the resistant NILs. A software package, namely Plant MetGenMAP^34^, was used for studying the pathways specific to each NILs in detail.

### Cluster and network analysis

SDEL common between the resistant NILs PB1 + *Pi9* 24 hpi and PB1+*Pi54* 72 hpi were clustered using k-means after calculating the Euclidean distance. Network analysis of proteins (corresponding to SDEL) common between the resistant NILs PB1 + *Pi9* 24 hpi and PB1 + *Pi54* 72 hpi was undertaken using STRING 10^35^. In the protein network, each circular node represents a protein (a specific number) that is encoded by the respective locus. The interconnecting lines between nodes represent the sources of association between two proteins. These sources were colour-coded as follows: black, co-expression; pink, experimental data; green, text mining; and blue, homology. The total score represents the confidence of each association between proteins.

### Real-time PCR validation

A real-time PCR was set up to validate the SDEL common between the resistant NILs PB1 + *Pi9* and PB1 + *Pi54*. A high capacity cDNA synthesis kit (ABI) was used for preparing cDNA. The qRT-PCR reaction was set up in a LightCycler® 480 II PCR system (Roche) (a volume of 20 μL containing 10 μL SYBR Green I Master, 1 μL reverse primer, 1 μL forward primer, 3 μL RNAase free water, and 5 μL cDNA template diluted in a ratio of 1:2). The primers used, listed in Supplementary Table 9, were designed using PrimerQuest (Integrated DNA Technologies), and GAPDH was used as the reference gene. The efficiency of the qRT-PCR was calculated in both target and control samples, and the fold change was calculated using 2^−ΔΔCT^ method.

## Conclusions

We analysed transcriptomes of three resistant NILs along with its susceptible recurrent parent PB1 and identified six SDEL commonly induced in all the three blast-resistant NILs (PB1 + *Pi9*, PB1 + *Pi54*, and PB1 + *Pi1*) 24 hpi with *M. oryzae*. Co-expression network analysis of the commonly induced SDEL provided protein-level evidence of the common network found in rice lines against *M. oryzae*. Through pathway analysis, gene set enrichment, and co-expression analysis of SDEL unique to the resistant NILs PB1 + *Pi9* 24 hpi, PB1 + *Pi1* 24 hpi, and PB1 + *Pi54* 24 and 72 hpi, the mechanisms of broad-spectrum resistance common to PB1 + *Pi9* and PB1 + *Pi54* were identified. These findings suggest important candidate genes for breeding rice varieties with broad-spectrum resistance to *M. oryzae*. Two novel significant (FDR adjusted *p* value ≤ 0.05) differentially expressed alternative splice variants common between the resistant NILs PB1 + *Pi9* 24 hpi and PB1 + *Pi54* 72 hpi offer a greater understanding of transcriptional-level changes involved in broad-spectrum resistance. A comparison of the resistance mechanisms specific to each NIL showed that, in total, gene *Pi9* contributes 52% (876 SDEL) to the level of resistance against *M. oryzae*, gene *Pi54* contributes 43% (725 SDEL), and gene *Pi1* contributes 5% (82 SDEL). By pyramiding *Pi9* and *Pi54*, breeders can develop a variety that offers a strong broad-spectrum resistance to the blast disease of rice.

## Supplementary information

Supplementary Figure And Table
