# Supplementary material for: Deciphering signalling network in broad spectrum Near Isogenic Lines of rice resistant to Magnaporthe oryzae
Source: Sci Rep. 2019 Nov 15;9:16939. doi: 10.1038/s41598-019-50990-8 (PMC6858299; doi:10.1038/s41598-019-50990-8)
Supplement: Supplementary file 1 — Supplementary Figure And Table [file 41598_2019_50990_MOESM1_ESM.doc]

**Deciphering signalling network in broad spectrum Near Isogenic Lines of rice resistant to *Magnaporthe oryzae***

Priyanka Jain ^1^, Himanshu Dubey^1^, Pankaj Kumar Singh ^2^, Amolkumar U. Solanke^1^, Ashok K. Singh ^3^ and T. R. Sharma^1, 2*^

^1^ICAR-National Institute for Plant Biotechnology, Pusa Campus, New Delhi-110012, India

^2^ National Agri-Food Biotechnology Institute, Mohali, Punjab, India

^3^ ICAR-Indian Agricultural Research Institute, New Delhi-110012, India

**SUPPLEMENTARY INFO**

**Supplementary Figure 1**: Co-expression network of proteins corresponding to SDEL commonly induced in all three resistant NILs (PB1+*Pi9* 24 hpi, PB1+*Pi1* 24 hpi and PB1+*Pi54* 24 hpi) but absent in susceptible control PB1 24 hpi after *M. oryzae* infection.

**Supplementary Figure 2**: Pathway Analysis of SDEL specific to PB1+*Pi1 (A); PB1*+*Pi9* (B) and PB1+*Pi54 (C)* NILs.

**Supplementary Figure 3**: Metabolism Overview of SDEL specific to PB1+*Pi1 (A);* PB1+*Pi9* (B) and PB1+*Pi54* (C) NILs.

**Supplementary Figure 4**: Common pathways of SDEL specific to PB1+*Pi9* 24 hpi and PB1+*Pi54* 72 hpi NILs, respectively and pathways specific to each NIL PB1+*Pi9,* PB1+*Pi1* and PB1+*Pi54* respectively.

**Supplementary Table 1:** Phenotyping reaction* of different NILs against six *M. oryzae* strain.

**Supplementary Table 2**: The number of left and right reads mapped in all three resistant NIL and susceptible control

**Supplementary Table 3:** List of common SDEL between resistant NIL PB1+*Pi9* 24 hpi PB1+*Pi1* 24 hpi & PB1+*Pi54* 24hpi but absent in PB1.

**Supplementary Table 4:** Co-expression network of genes common in *Pi9* 24 hpi, *Pi1* 24 hpi & *Pi54* 24 hpi

**Supplementary Table 5**: The number of upregulated and downregulated genes among the unique SDEL in each NIL

**Supplementary Table 6:** GO enrichment of unique SDEL in PB1+*Pi9 24 hpi*

**Supplementary Table 7:** GO enrichment of unique SDEL in PB1+*Pi54 72 hpi.*

**Supplementary Table 8:** Details of SDEL common between PB1+*Pi9* 24 hpi and PB1+ *Pi54* 72 hpi

**Supplementary Table 9:** Details of SDEL and primer used for real time PCR.

**Supplementary Table 10:** Details of clustering between loci common between PB1+*Pi9* 24 hpi and PB1+*Pi54* 72 hpi.

**Supplementary Table 11**: Details of co-expression network formed by unique SDEL common between PB1+*Pi9* 24 hpi and PB1+*Pi54* 72 hpi.

**Supplementary Table 12:** Pathway found in co-expression network of proteins corresponding to the significant (log_2_fold change ≥ 2) loci common between resistant NILs PB1+*Pi9* 24 hpi and PB1+*Pi54* 72 hpi.

**Supplementary Figure 1**

**
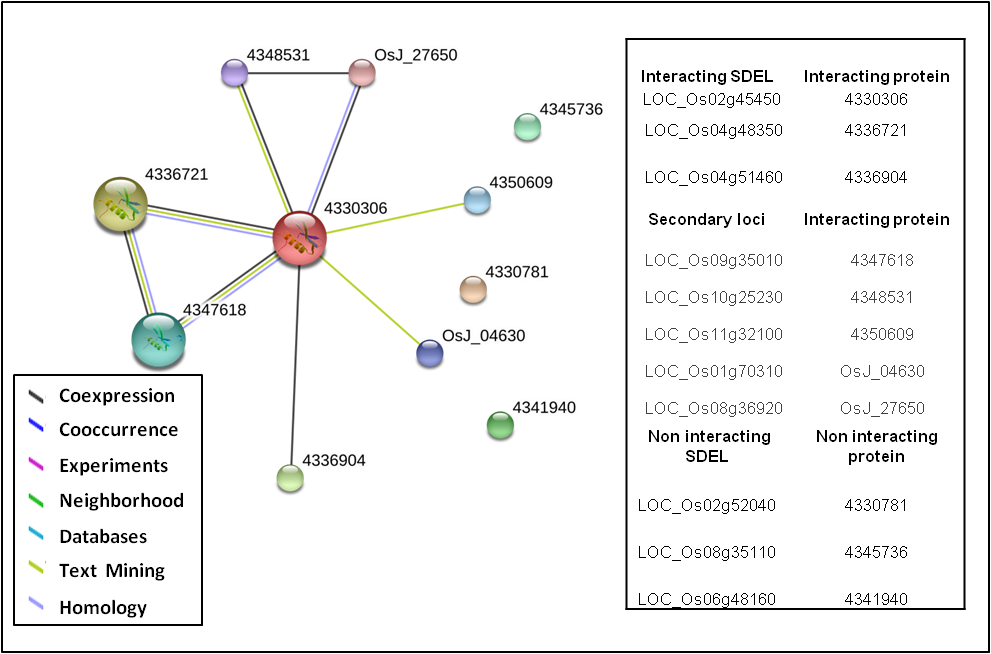
**

**Supplementary Figure 2**


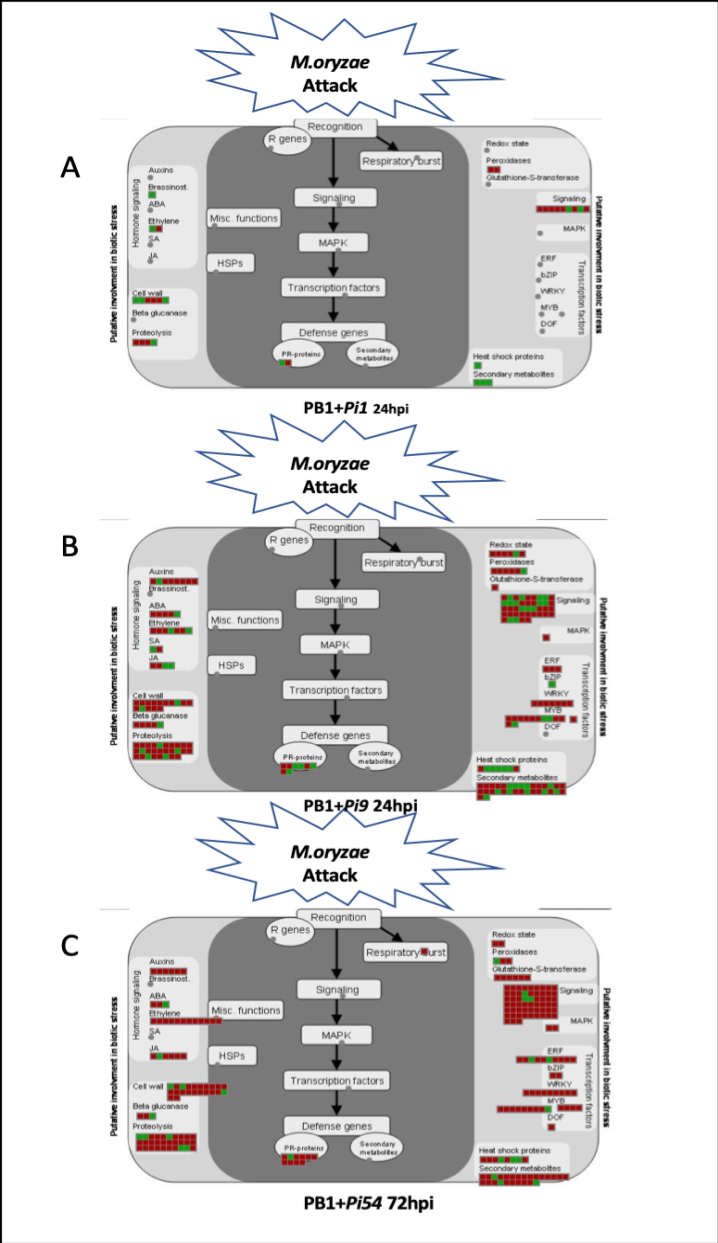


**Supplementary Figure 3**


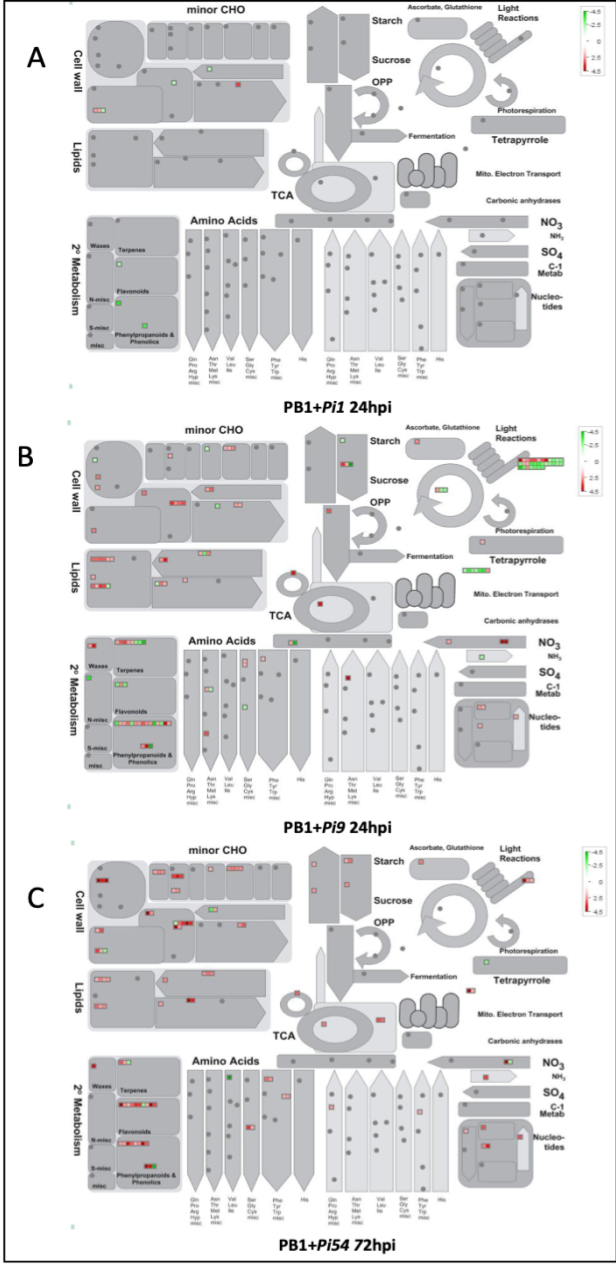


**Supplementary Figure 4**

**PATHWAY COMMON BETWEEN PB1+*Pi9* 24hpi & PB1+*Pi54* 72hpi**

**Jasmonic Acid Biosynthesis Salicylate Biosynthesis**

**
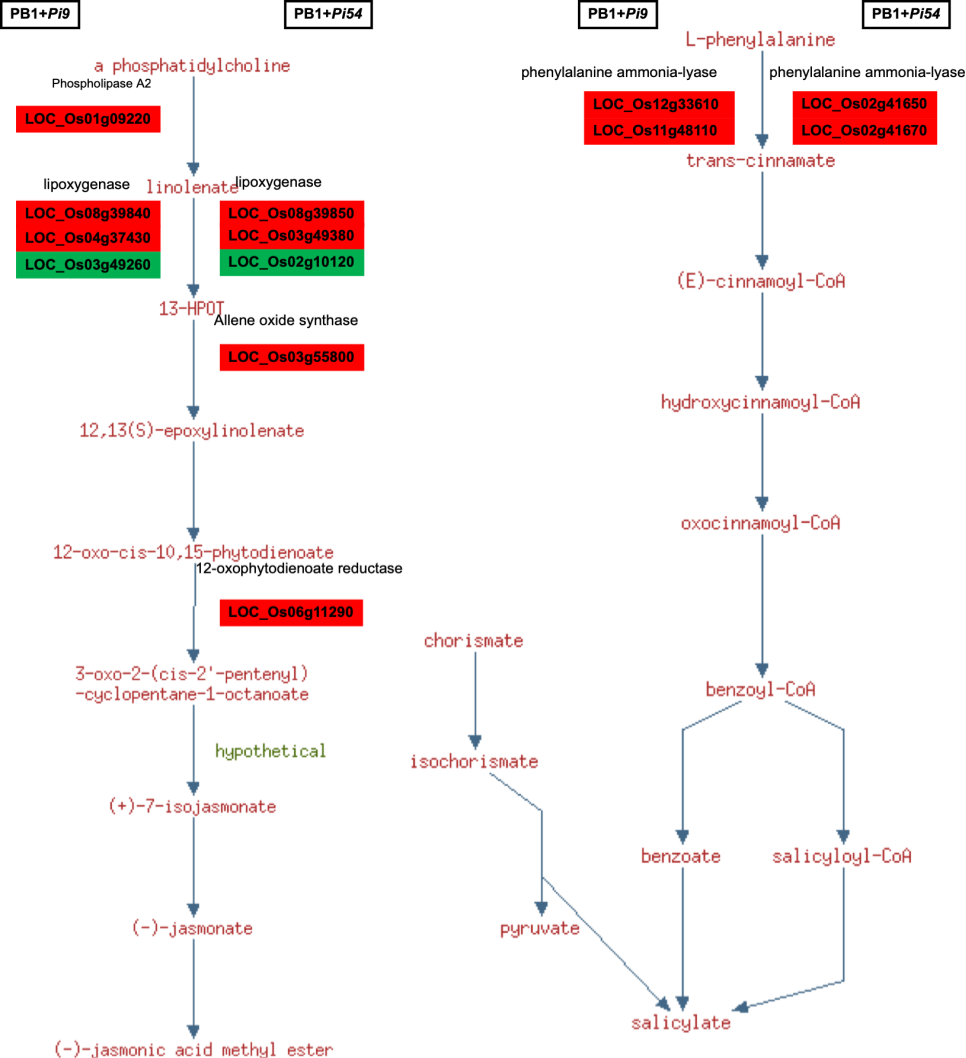
**

**13-LOX and 13-HPL pathway**

**
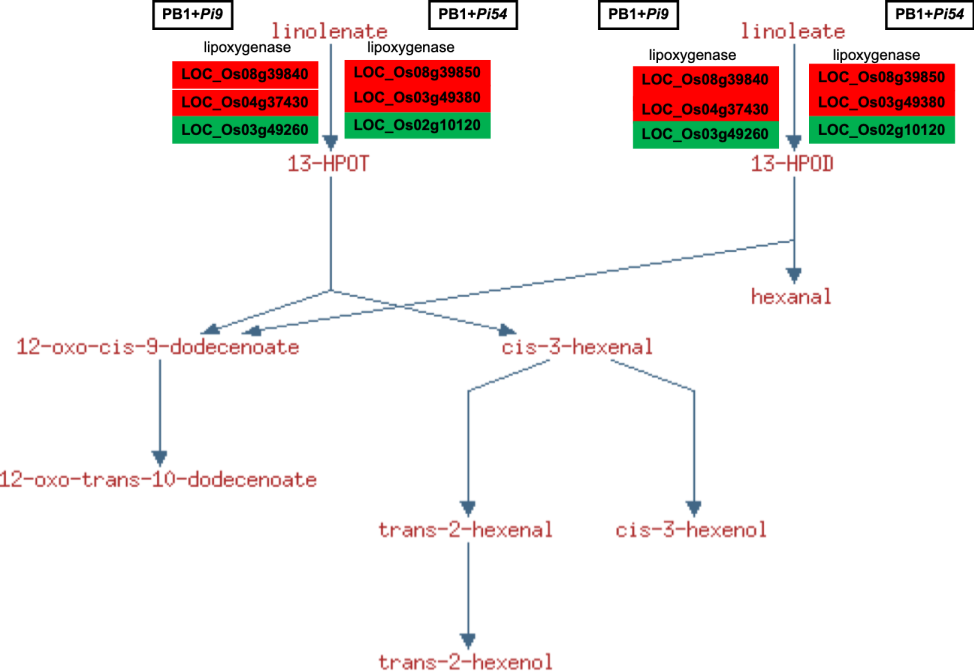
**

**divinyl ether biosynthesis II phenylpropanoid biosynthesis,initial reaction**

**
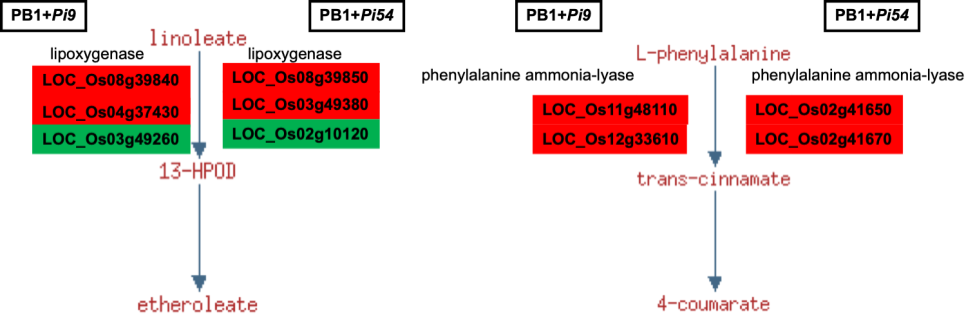
**

**Suberin Biosynthesis**


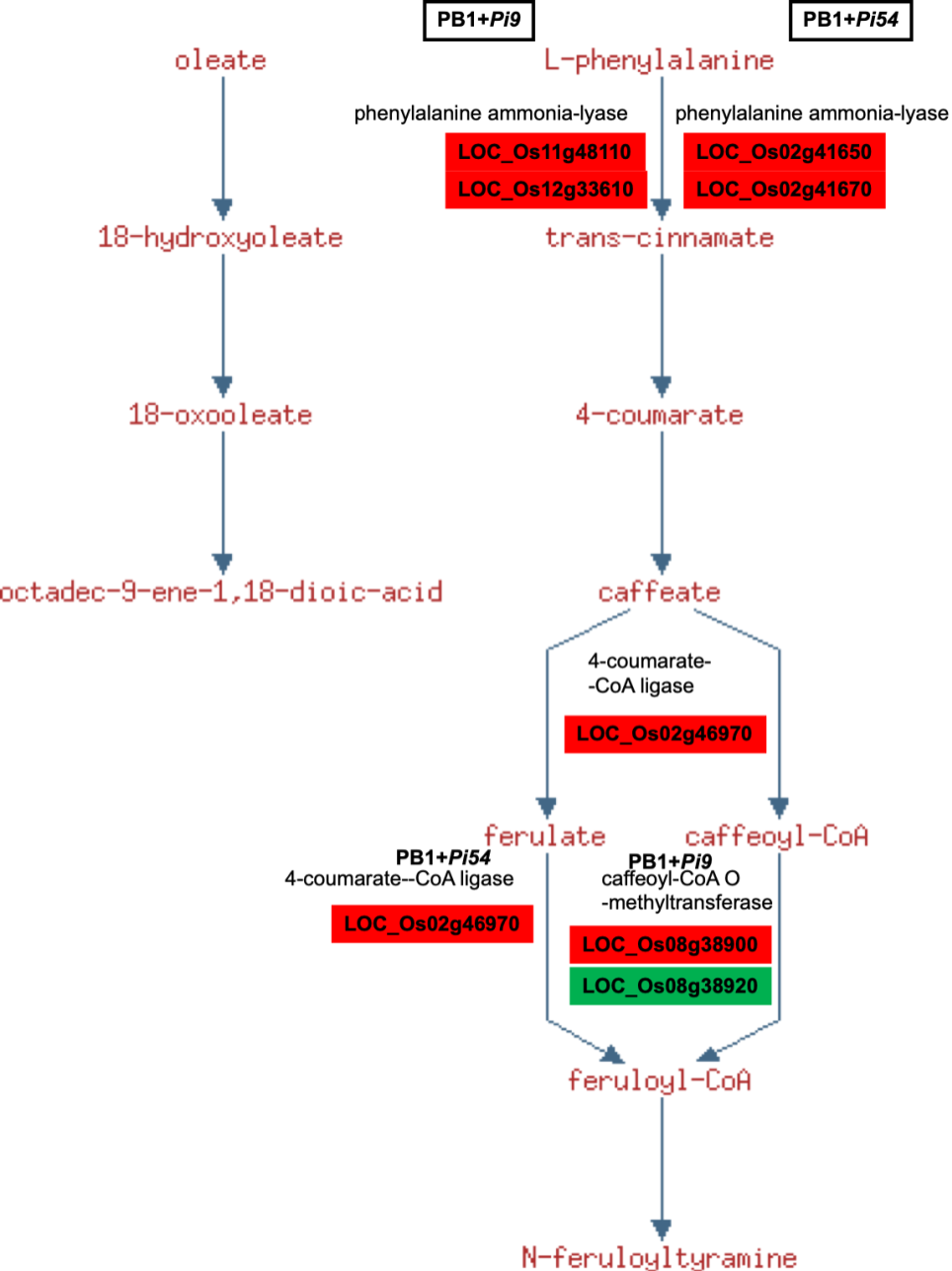


**PATHWAY SPECIFIC TO PB1+*Pi9***

**Cellulose biosynthesis**


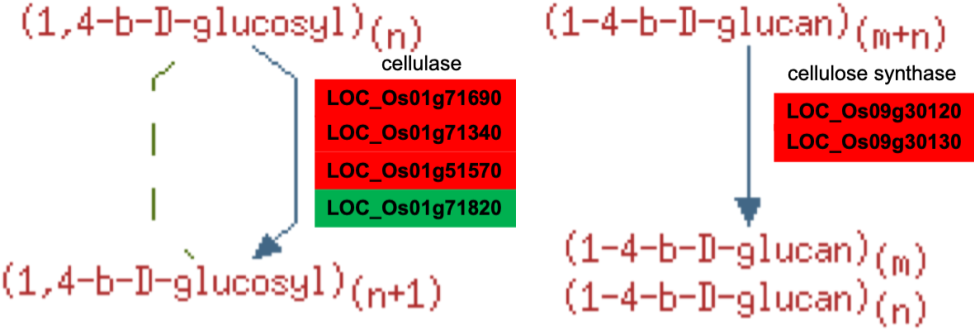


**UDP-D-glucuronate biosynthesis**  **UDP-D-xylose biosynthesis**


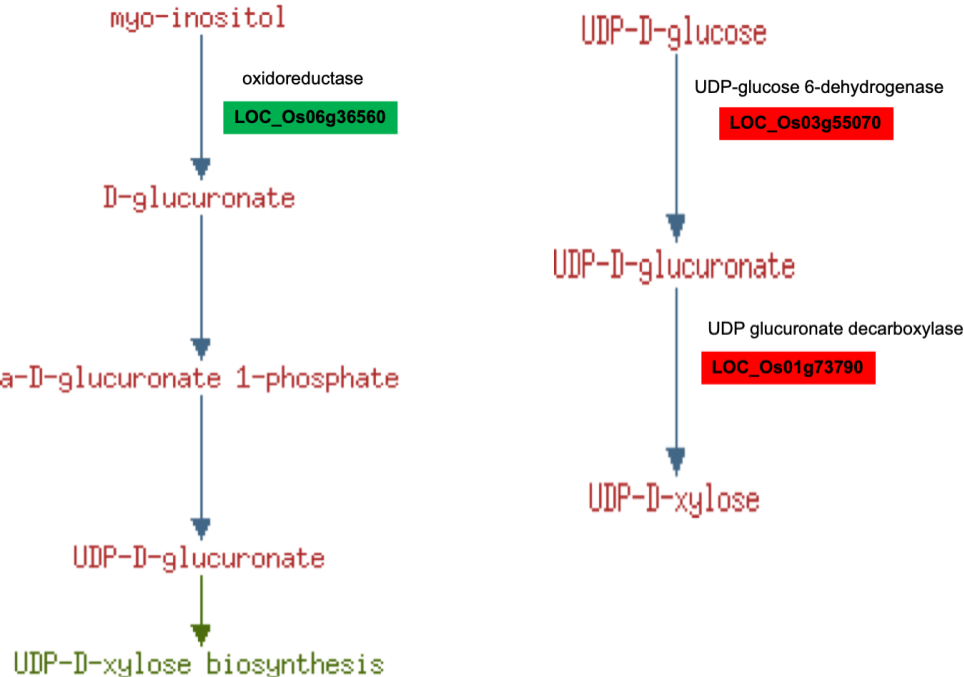


**salvage pathways of purine nucleosides II**


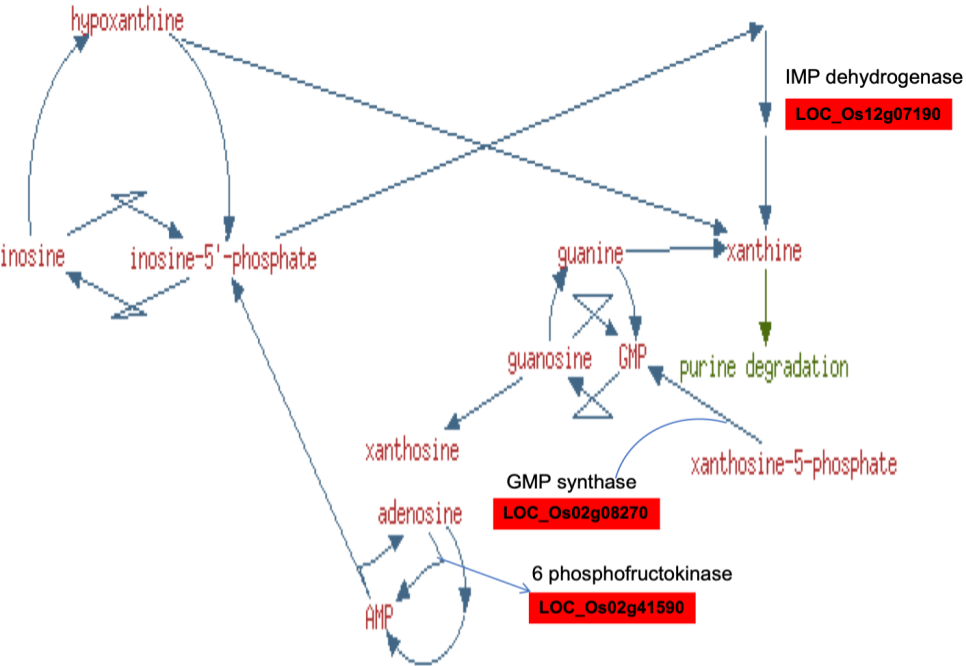


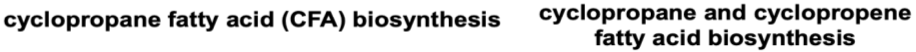


**
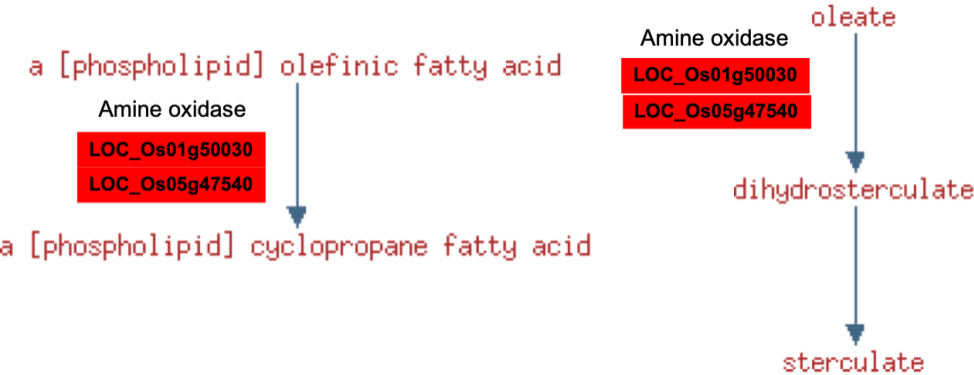
**

**
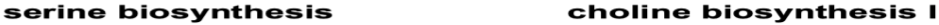
**


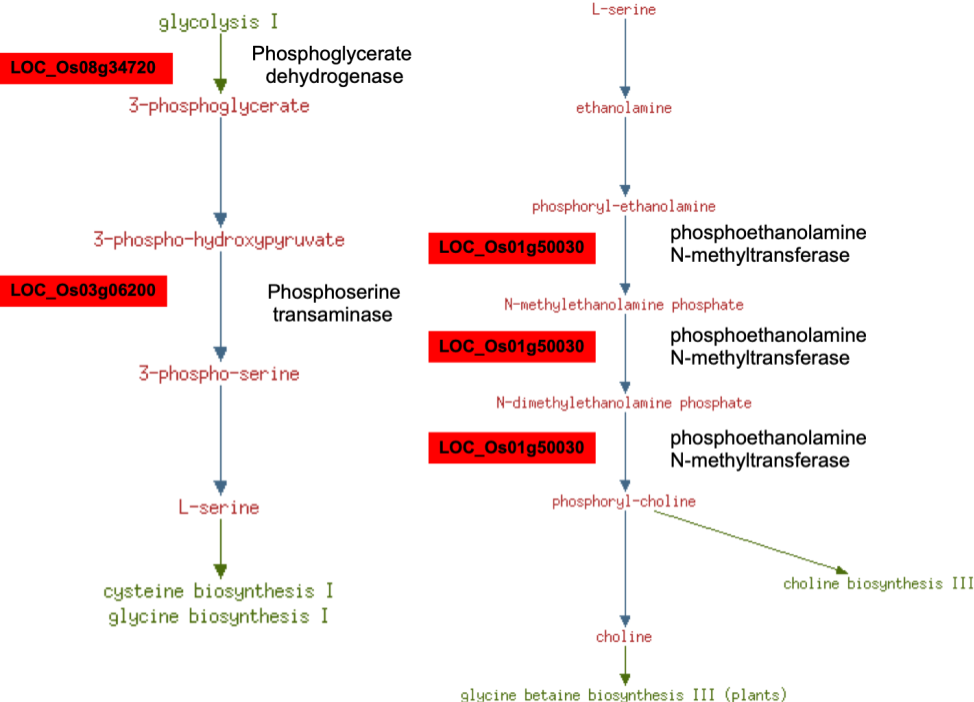


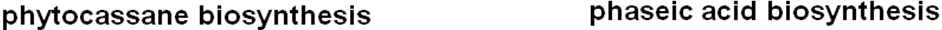


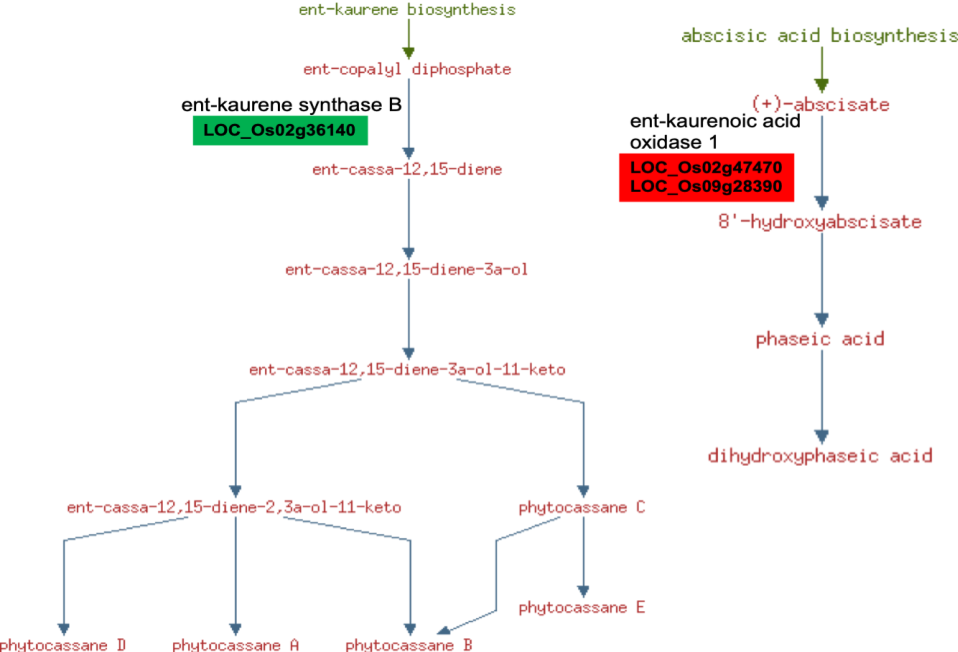


**Phenylpropanoid biosynthesis**


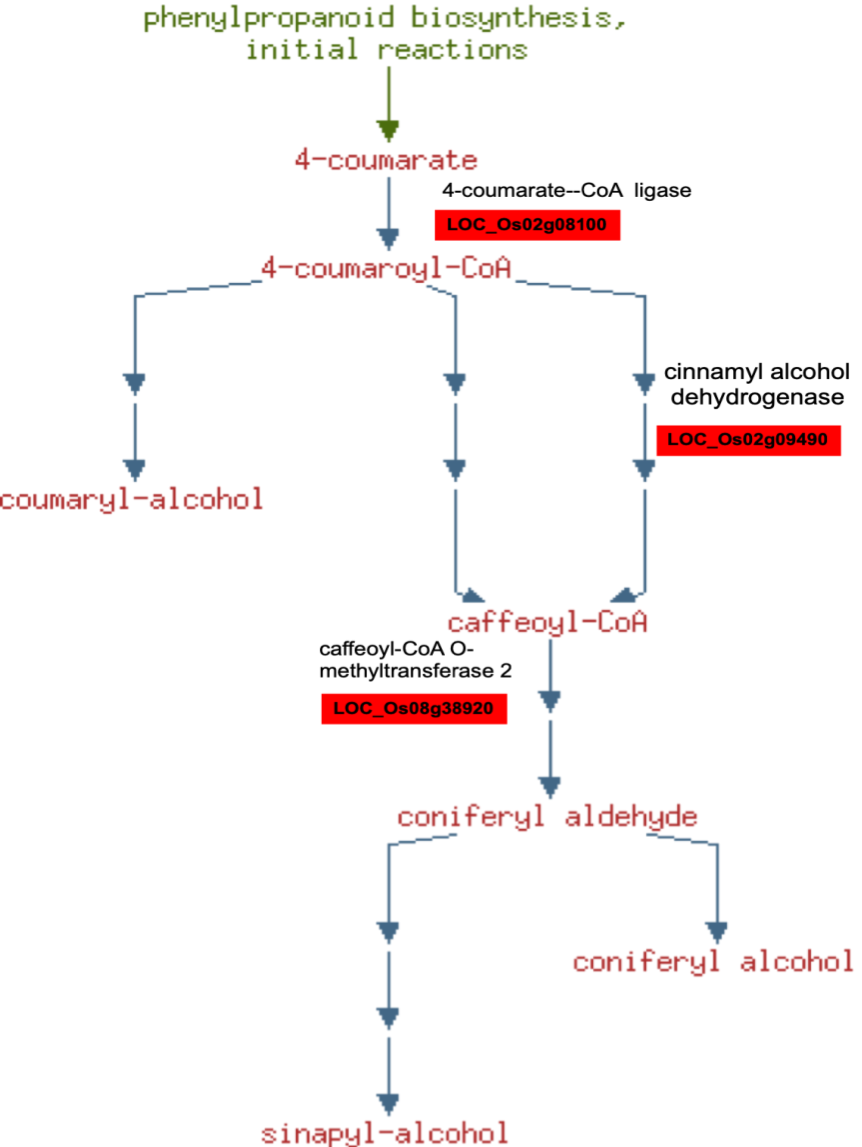


**PATHWAY SPECIFIC TO PB1+*Pi54* 72hpi**

**GDP-mannose metabolism**

***
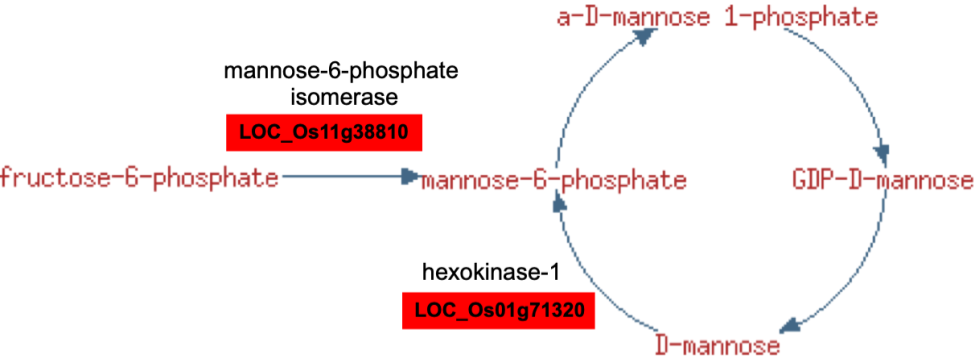
***

**
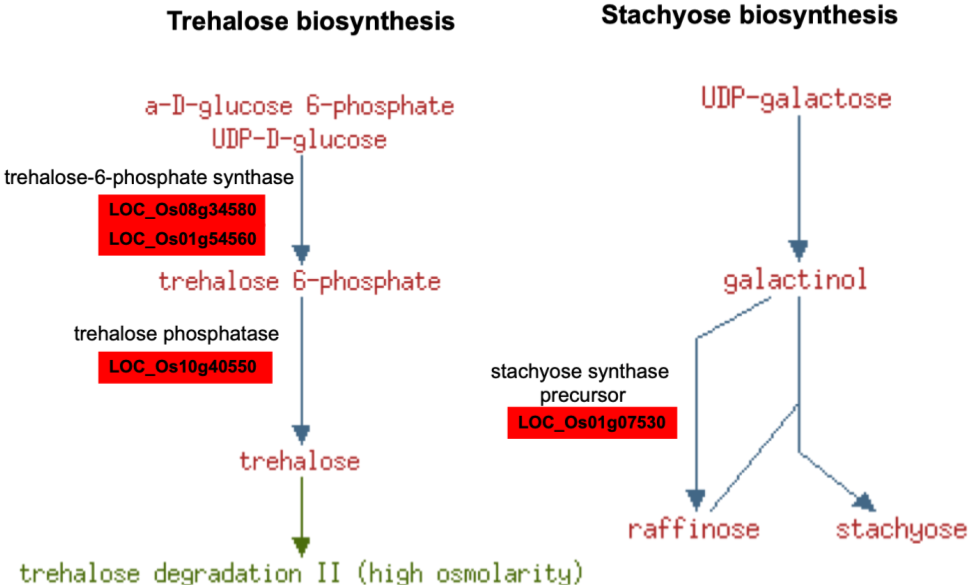
**

**
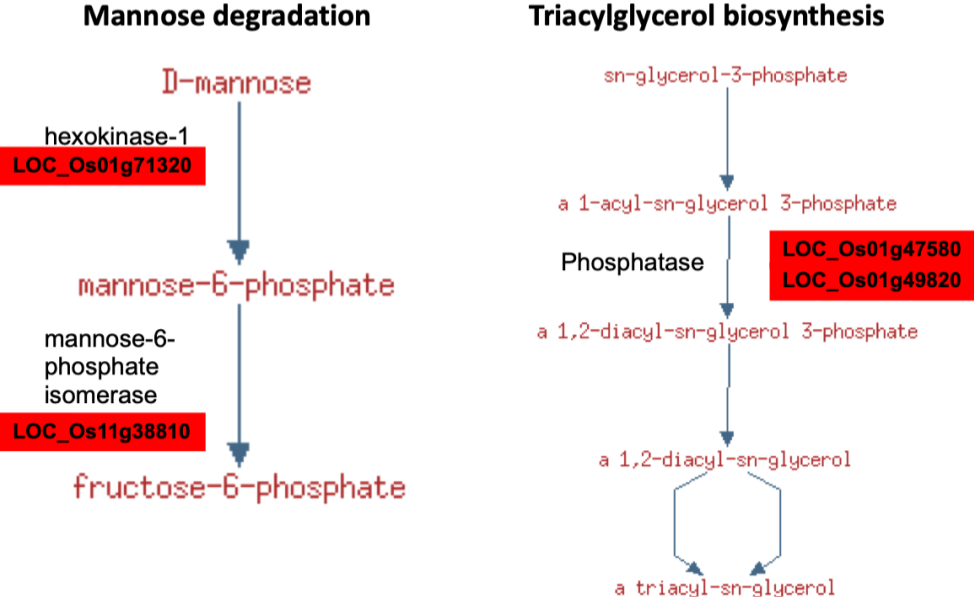
**

**
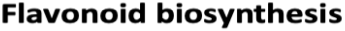

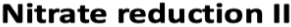
**

**
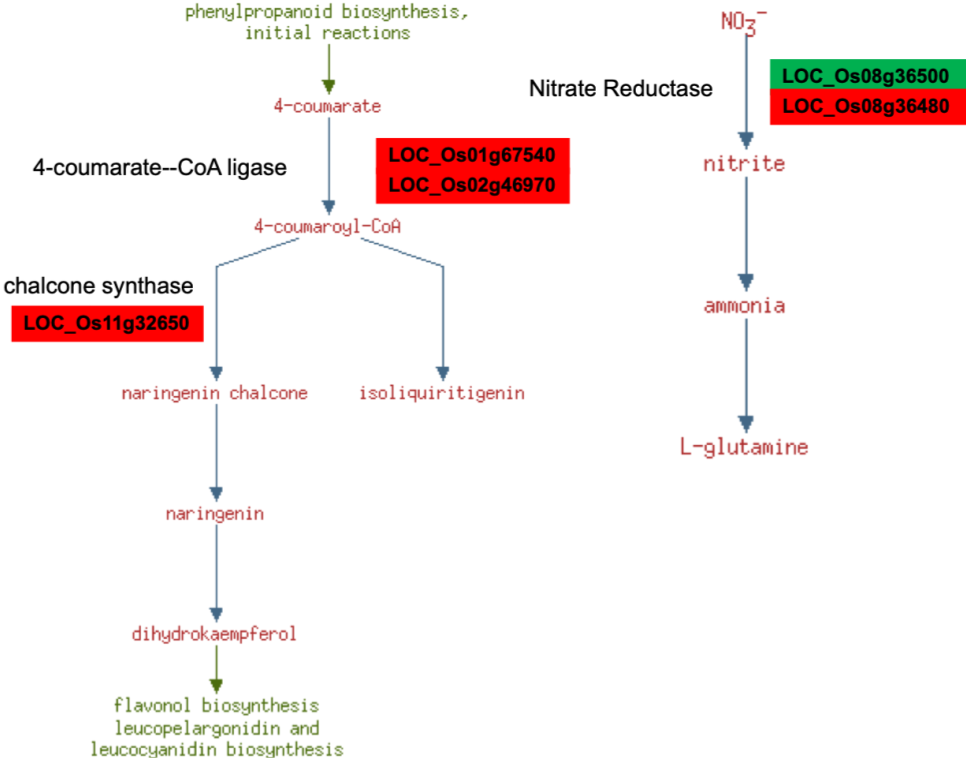
**

**PATHWAY SPECIFIC TO PB1+*Pi1* 24hpi**

**
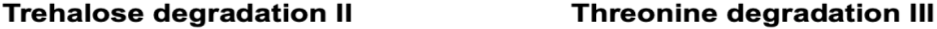
**

**
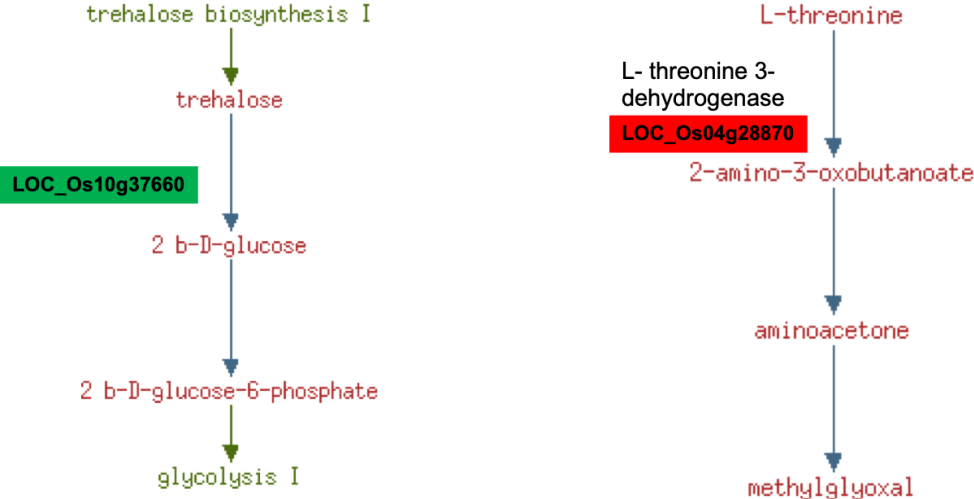
**

**
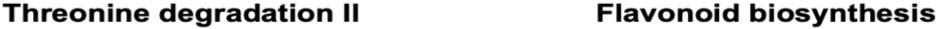
**

**
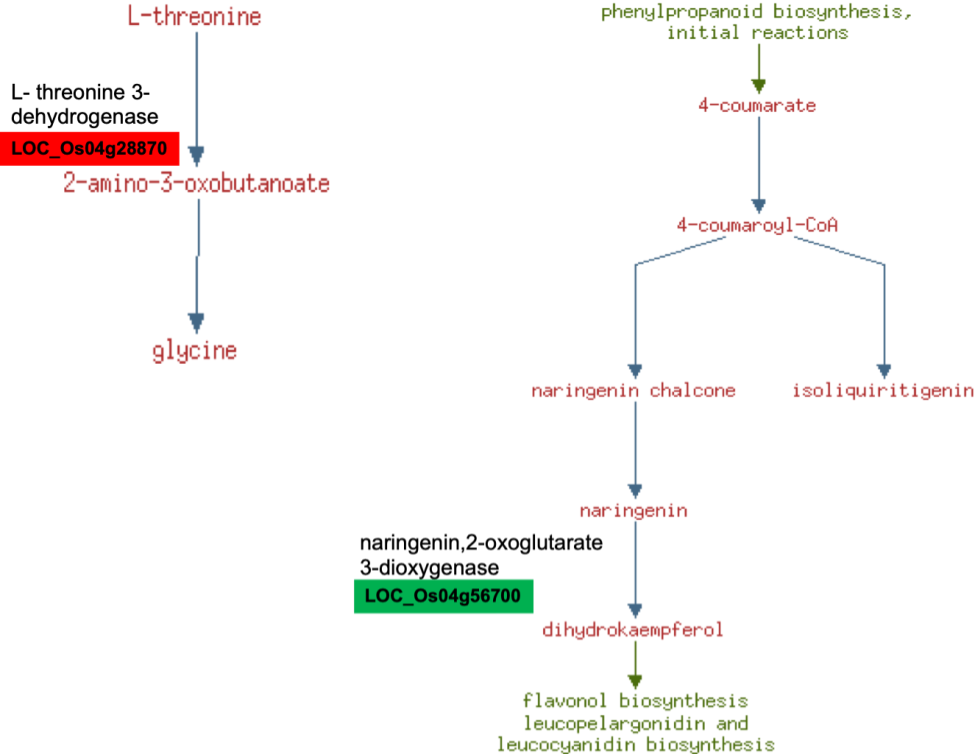
**

**
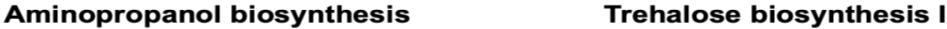
**

**
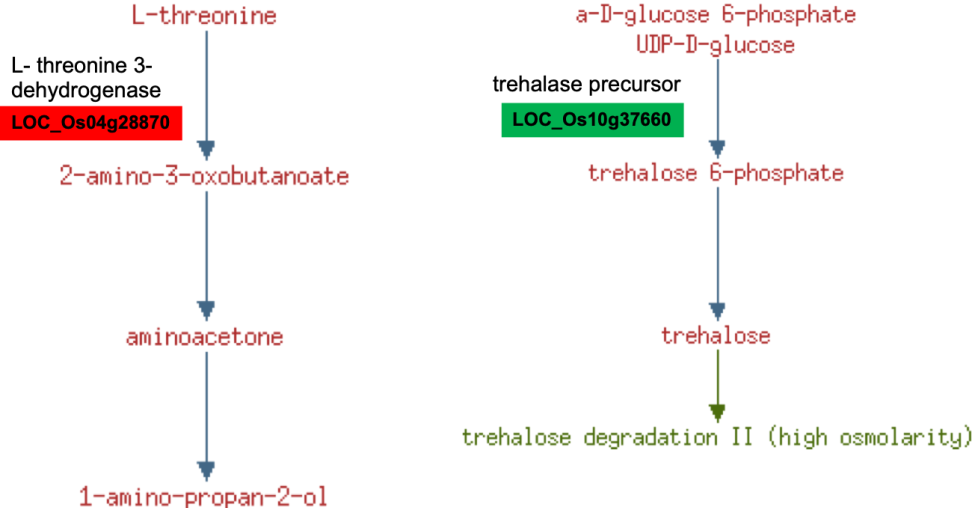
**

**Supplementary Table 1:** Phenotyping reaction* of different NILs against six *M. oryzae* strain

| **Near Isogenic Line** | **Mo-nwi-53** | **Sheeru3** | **Moei-11** | **Dehradun** | **Pusa basmati** | **MG-91** |
| --- | --- | --- | --- | --- | --- | --- |
| PB1+*Pi9* | 0 | 1 | 3 | 2 | 1 | 1 |
| PB1+*Pi54* | 1 | 2 | 3 | 3 | 1 | 3 |
| PB1+*Pi1* | 2 | 2 | 3 | 3 | 1 | 2 |
| PB1+*Pita* | 2 | 3 | 4 | 5 | 1 | 0 |
| PB1+*Pi5* | 0 | 4 | 4 | 5 | 1 | 0 |
| PB1+*Pib* | 1 | 4 | 4 | 4 | 1 | 0 |
| PB1+*Piz5* | 4 | 4 | 5 | 4 | 1 | 1 |
| PB1 | 5 | 5 | 5 | 5 | 3 | 4 |
| HR12  (Highly susceptible control) | 5 | 5 | 5 | 5 | 5 | 4 |

*Reaction was recorded on rice seedlings by using (0- 5) disease rating scale (Mackill and Bonman et al., 1992)

**Supplementary Table 2**: The number of left and right reads mapped in all three resistant NIL and susceptible control

| **Name of Sample** | **Left reads** |  |  | **Right reads** |  |  |
| --- | --- | --- | --- | --- | --- | --- |
|  | **Input** | **Mapped** | **Percentage mapped** | **Input** | **mapped** | **Percentage mapped** |
|  |  |  |  |  |  |  |
| Pi9_Mock_24hpi_BR1 | 32842898 | 30122237 | 91.70% | 32842898 | 29984065 | 91.30% |
|  |  |  |  |  |  |  |
| Pi9_Mock_24hpi_BR2 | 23278335 | 21400851 | 91.90% | 23278335 | 21298274 | 91.50% |
|  |  |  |  |  |  |  |
| Pi9_Mock_24hpi_BR3 | 32542514 | 29771254 | 91.50% | 32542514 | 29625170 | 91% |
|  |  |  |  |  |  |  |
| Pi9_Trtd_24hpi_BR1 | 27337405 | 24953691 | 91.30% | 27337405 | 24825875 | 90.80% |
|  |  |  |  |  |  |  |
| Pi9_Trtd_24hpi_BR2 | 24447346 | 22314390 | 91.30% | 24447346 | 22189970 | 90.80% |
|  |  |  |  |  |  |  |
| Pi9_Trtd_24hpi_BR3 | 22854945 | 20990594 | 91.80% | 22854945 | 20889392 | 91.40% |
|  |  |  |  |  |  |  |
| Pi1_Mock_24hpi_BR1 | 38471007 | 33891526 | 88.10% | 38471007 | 33215624 | 86.30% |
|  |  |  |  |  |  |  |
| Pi1_Mock_24hpi_BR2 | 30795286 | 27200209 | 88.30% | 30795286 | 26550283 | 86.20% |
|  |  |  |  |  |  |  |
| Pi1_Mock_24hpi_BR3 | 26262614 | 23022050 | 87.70% | 26262614 | 22597924 | 86.00% |
|  |  |  |  |  |  |  |
| Pi1_Trtd__24hpi_BR1 | 29907437 | 26305208 | 88.00% | 29907437 | 25718536 | 86.00% |
|  |  |  |  |  |  |  |
| Pi1_Trtd_24hpi_BR2 | 33628254 | 29652424 | 88.20% | 33628254 | 28998235 | 86.20% |
|  |  |  |  |  |  |  |
| Pi1_Trtd_24hpi_BR3 | 24553893 | 21687034 | 88.30% | 24553893 | 21203633 | 86.40% |
|  |  |  |  |  |  |  |
| Pi54_Mock_24hpi_BR1 | 36802238 | 32467816 | 88.20% | 36802238 | 31942439 | 86.80% |
|  |  |  |  |  |  |  |
| Pi54_Mock_24hpi_BR2 | 38313252 | 33846783 | 88.30% | 38313252 | 33308777 | 86.90% |
|  |  |  |  |  |  |  |
| Pi54_Mock_24hpi_BR3 | 42129438 | 36934454 | 87.70% | 42129438 | 36311782 | 86.20% |
|  |  |  |  |  |  |  |
| Pi54_Trtd_24hpi_BR1 | 38948992 | 34308370 | 88.10% | 38948992 | 33812769 | 86.80% |
|  |  |  |  |  |  |  |
| Pi54_Trtd_24hpi_BR2 | 37357435 | 33154364 | 88.70% | 37357435 | 32445884 | 86.90% |
|  |  |  |  |  |  |  |
| Pi54_Trtd_24hpi_BR3 | 35435427 | 31253556 | 88.20% | 35435427 | 30636172 | 86.50% |
|  |  |  |  |  |  |  |
| Pi54_Mock_72hpi_BR1 | 55936524 | 48948119 | 87.50% | 55936524 | 47684283 | 85.20% |
|  |  |  |  |  |  |  |
| Pi54_Mock_72hpi_BR2 | 28693435 | 25347452 | 88.30% | 28693435 | 24696627 | 86.10% |
|  |  |  |  |  |  |  |
| Pi54_Mock_72hpi_BR3 | 23817524 | 21028524 | 88.30% | 23817524 | 20531957 | 86.20% |
|  |  |  |  |  |  |  |
| Pi54_Trtd_72hpi_BR1 | 28504678 | 25192498 | 88.40% | 28504678 | 24558573 | 86.20% |
|  |  |  |  |  |  |  |
| Pi54_Trtd_72hpi_BR2 | 27223996 | 24204657 | 88.90% | 27223996 | 23324366 | 85.70% |
|  |  |  |  |  |  |  |
| Pi54_Trtd_72hpi_BR3 | 26642036 | 23684794 | 88.90% | 26642036 | 23077445 | 86.60% |
|  |  |  |  |  |  |  |
| PB1_Mock_24hpi_BR1 | 24592987 | 22558468 | 91.70% | 24592987 | 22582754 | 91.80% |
|  |  |  |  |  |  |  |
| PB1_Mock_24hpi_BR2 | 22876122 | 21015876 | 91.90% | 22876122 | 21045390 | 92% |
|  |  |  |  |  |  |  |
| PB1_Mock_24hpi_BR3 | 26671361 | 24520761 | 91.90% | 26671361 | 24544740 | 92% |
|  |  |  |  |  |  |  |
| PB1_Trtd_24hpi_BR1 | 29547233 | 27360504 | 92.60% | 29547233 | 27383075 | 92.70% |
|  |  |  |  |  |  |  |
| PB1_Trtd_24hpi_BR2 | 23520141 | 21711289 | 92% | 23520141 | 21738632 | 92% |
|  |  |  |  |  |  |  |
| PB1_Trtd_24hpi_BR3 | 25100931 | 23170690 | 92% | 25100931 | 23195473 | 92% |
|  |  |  |  |  |  |  |
| PB1_Mock_72hpi_BR1 | 33884800 | 29590551 | 87.30% | 33884800 | 28931896 | 85.40% |
|  |  |  |  |  |  |  |
| PB1_Mock_72hpi_BR2 | 35859307 | 31416068 | 87.60% | 35859307 | 30251617 | 84.40% |
|  |  |  |  |  |  |  |
| PB1_Mock_72hpi_BR3 | 33541382 | 28991761 | 86.40% | 33541382 | 28376429 | 84.60% |
|  |  |  |  |  |  |  |
| PB1_Trtd_72hpi_BR1 | 36104367 | 31794231 | 88.10% | 36104367 | 31014517 | 85.90% |
|  |  |  |  |  |  |  |
| PB1_Trtd_72hpi_BR2 | 32531272 | 28724789 | 88.30% | 32531272 | 28059940 | 86.30% |
|  |  |  |  |  |  |  |
| PB1_Trtd_72hpi_BR3 | 30325325 | 26656317 | 87.90% | 30325325 | 26034279 | 85.80% |

**Supplementary Table 3:** List of common SDEL between resistant NIL PB1+*Pi9* 24hpi PB1+*Pi1* 24hpi & PB1+*Pi54* 24hpi but absent in PB1

| **SDEL** | **Functional Annotation** | **PB1+*Pi9* 24hpi** | **PB1+*Pi54* 24hpi** | **PB1+*Pi1* 24hpi** |
| --- | --- | --- | --- | --- |
| LOC_Os02g45450 | TF | 6.13321 | -2.98082 | 4.20626 |
| LOC_Os08g35110 | Hormone | 5.05432 | -2.62096 | 2.6845 |
| LOC_Os04g48350 | TF | 4.3448 | -3.02911 | 4.29771 |
| LOC_Os04g51460 | Glucosyl hydrolase | 4.3248 | 2.67003 | -2.63224 |
| LOC_Os02g52040 | Oxidative stress | 3.83664 | 3.0767 | -2.79932 |
| LOC_Os06g48160 | Glucosyl hydrolase | 2.90755 | -4.6983 | 2.77909 |

**Supplementary Table 4:** Co-expression network of genes common in *Pi9* 24hpi, *Pi1* 24hpi & *Pi54* 24hpi

| #node1 | node2 | node1_external_id | node2_external_id | neighborhood_on_chromosome | gene_fusion | phylogenetic_cooccurrence | Homology | Coexpression | experimentally_determined_interaction | database_annotated | automated_textmining | combined_  score |
| --- | --- | --- | --- | --- | --- | --- | --- | --- | --- | --- | --- | --- |
| 4347618 | 4E+06 | 39947.LOC_Os09g35010.1 | 39947.LOC_Os02g45450.1 | 0 | 0 | 0 | 0.854 | 0.937 | 0 | 0 | 0.493 | 0.941 |
| 4348531 | OsJ_27650 | 39947.LOC_Os10g25230.1 | 39947.LOC_Os08g36920.1 | 0 | 0 | 0 | 0 | 0.913 | 0 | 0 | 0 | 0.913 |
| 4350609 | 4E+06 | 39947.LOC_Os11g32100.1 | 39947.LOC_Os02g45450.1 | 0 | 0 | 0 | 0 | 0 | 0 | 0 | 0.758 | 0.758 |
| 4330306 | OsJ_04630 | 39947.LOC_Os02g45450.1 | 39947.LOC_Os01g70310.1 | 0 | 0 | 0 | 0 | 0 | 0 | 0 | 0.758 | 0.758 |
| 4336721 | 4E+06 | 39947.LOC_Os04g48350.1 | 39947.LOC_Os02g45450.1 | 0 | 0 | 0 | 0.972 | 0.697 | 0 | 0 | 0.494 | 0.701 |
| 4348531 | 4E+06 | 39947.LOC_Os10g25230.1 | 39947.LOC_Os02g45450.1 | 0 | 0 | 0 | 0 | 0.304 | 0 | 0 | 0.498 | 0.635 |
| OsJ_27650 | 4E+06 | 39947.LOC_Os08g36920.1 | 39947.LOC_Os02g45450.1 | 0 | 0 | 0 | 0.673 | 0.599 | 0 | 0 | 0 | 0.599 |
| 4347618 | 4E+06 | 39947.LOC_Os09g35010.1 | 39947.LOC_Os04g48350.1 | 0 | 0 | 0 | 0.878 | 0.427 | 0 | 0 | 0.496 | 0.46 |
| 4336904 | 4E+06 | 39947.LOC_Os04g51460.1 | 39947.LOC_Os02g45450.1 | 0 | 0 | 0 | 0 | 0.413 | 0 | 0 | 0 | 0.412 |

**Supplementary Table 5**: The number of upregulated and downregulated genes among the unique SDEL in each NIL

|  | **PB1+*Pi9***  **24hpi** | **PB1+*Pi1***  **24hpi** | **PB1+*Pi54* 24hpi** | **PB1+*Pi54 7*2hpi** |
| --- | --- | --- | --- | --- |
| No. of upregulated SDEL | 727 | 68 | 9 | 769 |
| No. of downregulated SDEL | 316 | 49 | 82 | 89 |
| Total SDEL | 1043 | 117 | 91 | 858 |

**Supplementary Table 6:** GO enrichment of unique SDEL in PB1+*Pi9 24hpi*

| **GO_acc** | **term_type** | **Term** | **Query**  **item** | **FDR** |
| --- | --- | --- | --- | --- |
| GO:0050896 | P | response to stimulus | 246 | 5.80E-09 |
| GO:0006950 | P | response to stress | 179 | 1.50E-08 |
| GO:0015979 | P | Photosynthesis | 27 | 7.90E-06 |
| GO:0009628 | P | response to abiotic stimulus | 111 | 0.00042 |
| GO:0009607 | P | response to biotic stimulus | 61 | 0.00061 |
| GO:0008152 | P | metabolic process | 514 | 0.0039 |
| GO:0009719 | P | response to endogenous stimulus | 75 | 0.0054 |
| GO:0019748 | P | secondary metabolic process | 30 | 0.0054 |
| GO:0006091 | P | generation of precursor metabolites and energy | 24 | 0.031 |
| GO:0030528 | F | transcription regulator activity | 84 | 0.0098 |
| GO:0003700 | F | transcription factor activity | 84 | 0.0098 |
| GO:0019825 | F | oxygen binding | 21 | 0.018 |
| GO:0009579 | C | Thylakoid | 51 | 4.00E-08 |
| GO:0030312 | C | external encapsulating structure | 55 | 0.00031 |
| GO:0005618 | C | cell wall | 53 | 0.00065 |
| GO:0005576 | C | extracellular region | 33 | 0.018 |
| GO:0009536 | C | Plastid | 142 | 0.048 |

**Supplementary Table 7:** GO enrichment of unique SDEL in PB1+*Pi54 72hpi*

| **GO_acc** | **term_type** | **Term** | **Queryitem** | **FDR** |
| --- | --- | --- | --- | --- |
| GO:0008152 | P | metabolic process | 499 | 1.40E-08 |
| GO:0050896 | P | response to stimulus | 223 | 1.40E-08 |
| GO:0009719 | P | response to endogenous stimulus | 88 | 5.50E-08 |
| GO:0006950 | P | response to stress | 151 | 1.30E-05 |
| GO:0009628 | P | response to abiotic stimulus | 107 | 2.00E-05 |
| GO:0019748 | P | secondary metabolic process | 34 | 2.20E-05 |
| GO:0009058 | P | biosynthetic process | 207 | 0.00042 |
| GO:0009607 | P | response to biotic stimulus | 56 | 0.00047 |
| GO:0009987 | P | cellular process | 441 | 0.003 |
| GO:0030528 | F | transcription regulator activity | 90 | 6.80E-06 |
| GO:0003700 | F | transcription factor activity | 90 | 6.80E-06 |
| GO:0003824 | F | catalytic activity | 354 | 1.90E-05 |
| GO:0016740 | F | transferase activity | 150 | 0.0016 |
| GO:0019825 | F | oxygen binding | 21 | 0.0026 |
| GO:0003677 | F | DNA binding | 100 | 0.0035 |

**Supplementary Table 8:** Details of SDEL common between PB1+*Pi9* 24 hpi and PB1+ *Pi54* 72 hpi

| **SDEL** | **Fold change of NIL PB1+*Pi9*_24hpi** | **Fold change of NIL**  **PB1+*Pi54*_72hpi** | **SDEL Name** | **SDEL Involved Process** | **SDEL Role** |
| --- | --- | --- | --- | --- | --- |
| LOC_Os01g64120 | 6.23682 | 2.73542 |  |  |  |
| LOC_Os07g03120 | 5.54212 | 2.5888 |  |  |  |
| LOC_Os01g55160 | 5.40103 | 2.96826 |  |  |  |
| LOC_Os03g50960 | 4.74142 | 5.58052 |  |  |  |
| LOC_Os05g50930 | 4.59257 | 3.73594 |  |  |  |
| LOC_Os12g39360 | 4.52345 | -2.09733 |  |  |  |
| LOC_Os01g16980 | 4.50624 | 4.14715 |  |  |  |
| LOC_Os12g18560 | 4.44624 | 2.01714 |  |  |  |
| LOC_Os10g42960 | 4.12387 | 2.74759 |  |  |  |
| LOC_Os01g17396 | 4.07819 | 2.16301 |  |  |  |
| LOC_Os02g57720 | 3.92771 | 3.4497 |  |  |  |
| LOC_Os01g12690 | 3.82672 | -2.33026 |  |  |  |
| LOC_Os01g06836 | 3.74864 | 7.15203 |  |  |  |
| LOC_Os09g26780 | 3.68759 | 2.95659 |  |  |  |
| LOC_Os05g12630 | 3.65176 | -2.20673 |  |  |  |
| LOC_Os05g12640 | 3.65176 | -2.20673 |  |  |  |
| LOC_Os10g41100 | 3.64876 | 2.01914 |  |  |  |
| LOC_Os01g06876 | 3.64833 | 7.15203 |  |  |  |
| LOC_Os01g06882 | 3.64833 | 7.15203 |  |  |  |
| LOC_Os02g01590 | 3.60012 | 2.08866 | Glycosyl hydrolase | Starch Degradtaion | converts of beta fructose to alpha D glucose |
| LOC_Os07g22930 | 3.49943 | 3.07817 | Starch synthase | Starch Biosynthesis | converts alpha D- glucose-1-phosphate to ADP-D-glucose |
| LOC_Os06g40170 | 3.41174 | 4.28504 |  |  |  |
| LOC_Os08g04800 | 3.36148 | 3.9203 |  |  |  |
| LOC_Os01g03940 | 3.29724 | 2.14699 |  |  |  |
| LOC_Os04g45730 | 3.16643 | 2.33569 |  |  |  |
| LOC_Os02g11070 | 3.12395 | 2.01109 | 3-ketoacyl-CoA synthase | FA biosynthesis & elongation | converts of an acetyl group to all acyl carrier protein |
| LOC_Os04g48840 | 3.0812 | 3.38706 |  |  |  |
| LOC_Os01g22010 | 3.06646 | 2.35927 | S-adenosylmethionine synthetase | Ethylene biosynthesis | converts of L-methionine to S-adenosyl L-methionine |
| LOC_Os06g11210 | 3.04133 | 2.69534 | 12-oxophytodienoate reductase | JA biosynthesis | converts 12-oxo-cis-10,15-phytodienoate to 3-oxo-2-(cis-2-pentyl)-cyclopentane-1-octanoate |
| LOC_Os10g37570 | 3.02655 | 2.9215 |  |  |  |
| LOC_Os09g25720 | 2.96377 | 2.20358 |  |  |  |
| LOC_Os06g36070 | 2.95561 | 2.52983 |  |  |  |
| LOC_Os09g38910 | 2.88872 | 3.89765 |  |  |  |
| LOC_Os08g14570 | 2.88613 | 3.39665 |  |  |  |
| LOC_Os01g44050 | 2.86709 | 2.26886 |  |  |  |
| LOC_Os02g43540 | 2.82892 | 2.26003 |  |  |  |
| LOC_Os09g32100 | 2.82234 | 2.05787 |  |  |  |
| LOC_Os01g43460 | 2.81111 | 6.36797 |  |  |  |
| LOC_Os05g30500 | 2.78046 | 4.48571 |  |  |  |
| LOC_Os01g37750 | 2.78035 | 2.7241 |  |  |  |
| LOC_Os06g49470 | 2.77283 | 4.15717 |  |  |  |
| LOC_Os04g41970 | 2.76594 | 2.93546 |  |  |  |
| LOC_Os08g28710 | 2.70315 | 3.67174 |  |  |  |
| LOC_Os03g02514 | 2.69383 | 2.95308 |  |  |  |
| LOC_Os05g07870 | 2.68396 | 2.69092 |  |  |  |
| LOC_Os08g33720 | 2.67669 | 2.57307 |  |  |  |
| LOC_Os01g64110 | 2.67463 | 2.5674 |  |  |  |
| LOC_Os04g27790 | 2.65387 | 2.55889 |  |  |  |
| LOC_Os08g07620 | 2.61824 | 2.05912 |  |  |  |
| LOC_Os09g02710 | 2.59983 | 2.2522 |  |  |  |
| LOC_Os11g41870 | 2.59256 | 4.68803 |  |  |  |
| LOC_Os10g39680 | 2.57069 | 2.30028 |  |  |  |
| LOC_Os01g72610 | 2.57008 | 2.04623 |  |  |  |
| LOC_Os04g33390 | 2.52726 | 2.29903 |  |  |  |
| LOC_Os04g33060 | 2.50484 | 2.66517 |  |  |  |
| LOC_Os04g49950 | 2.49543 | 3.21126 |  |  |  |
| LOC_Os02g52780 | 2.48384 | 3.07346 |  |  |  |
| LOC_Os08g31850 | 2.46943 | 2.47784 |  |  |  |
| LOC_Os07g04560 | 2.46817 | 2.19185 |  |  |  |
| LOC_Os01g11160 | 2.45181 | 2.33773 |  |  |  |
| LOC_Os06g11240 | 2.39485 | 2.21811 | 12-oxophytodienoate reductase | JA biosynthesis | converts 12-oxo-cis-10,15-phytodienoate to 3-oxo-2-(cis-2-pentyl)-cyclopentane-1-octanoate |
| LOC_Os02g11870 | 2.39326 | 2.52687 |  |  |  |
| LOC_Os01g53090 | 2.37497 | 2.70327 |  |  |  |
| LOC_Os05g37190 | 2.36686 | 2.82924 |  |  |  |
| LOC_Os09g01960 | 2.34221 | 2.23513 |  |  |  |
| LOC_Os06g12560 | 2.33276 | 2.28592 |  |  |  |
| LOC_Os01g71310 | 2.31911 | 3.31414 |  |  |  |
| LOC_Os07g40290 | 2.31828 | 2.3902 |  |  |  |
| LOC_Os05g05640 | 2.29132 | 2.847 |  |  |  |
| LOC_Os08g38910 | 2.28348 | 2.68564 | caffeoyl-CoA O-methyl transferase | PPP biosynthesis | converts of caffeoyl-CoA to feruloyl-CoA |
| LOC_Os04g59330 | 2.27877 | 4.11786 |  |  |  |
| LOC_Os02g54060 | 2.26919 | 2.107 |  |  |  |
| LOC_Os01g18170 | 2.25407 | 3.26753 |  |  |  |
| LOC_Os09g06464 | 2.24457 | 2.34498 |  |  |  |
| LOC_Os09g28650 | 2.22341 | 4.76418 |  |  |  |
| LOC_Os02g11859 | 2.21429 | 2.2067 |  |  |  |
| LOC_Os07g02780 | 2.20892 | 2.30848 |  |  |  |
| LOC_Os04g32480 | 2.20383 | 4.64823 |  |  |  |
| LOC_Os03g59670 | 2.18808 | 2.8639 |  |  |  |
| LOC_Os03g50810 | 2.18096 | 2.68995 |  |  |  |
| LOC_Os01g69960 | 2.17596 | 2.03401 |  |  |  |
| LOC_Os02g48320 | 2.16612 | 2.16287 |  |  |  |
| LOC_Os09g20090 | 2.14264 | 2.87217 |  |  |  |
| LOC_Os07g42370 | 2.14094 | 2.4233 |  |  |  |
| LOC_Os09g04050 | 2.13754 | 2.97178 |  |  |  |
| LOC_Os01g60020 | 2.13745 | 3.30374 |  |  |  |
| LOC_Os05g47770 | 2.12191 | 3.23927 |  |  |  |
| LOC_Os09g39190 | 2.10553 | 3.07074 |  |  |  |
| LOC_Os07g38290 | 2.09337 | 2.40337 |  |  |  |
| LOC_Os04g51160 | 2.04805 | 2.70913 |  |  |  |
| LOC_Os04g46970 | 2.04642 | 2.7518 |  |  |  |
| LOC_Os02g13800 | 2.0439 | 4.12218 |  |  |  |
| LOC_Os03g01990 | 2.0437 | 2.19046 |  |  |  |
| LOC_Os09g38850 | 2.01882 | 2.13427 |  |  |  |
| LOC_Os01g62950 | 2.00652 | 4.79514 |  |  |  |
| LOC_Os12g13800 | -2.05702 | 4.90091 |  |  |  |
| LOC_Os12g13810 | -2.05702 | 4.90091 |  |  |  |
| LOC_Os03g04100 | -2.07349 | 3.15463 |  |  |  |
| LOC_Os02g52150 | -2.30324 | -2.31659 |  |  |  |
| LOC_Os08g39730 | -2.3494 | 3.55501 |  |  |  |
| LOC_Os03g01720 | -2.41145 | -2.49598 |  |  |  |
| LOC_Os03g13050 | -2.47531 | 2.01499 |  |  |  |
| LOC_Os04g31870 | -2.48067 | -2.18292 |  |  |  |
| LOC_Os01g04620 | -2.53338 | 2.68618 |  |  |  |
| LOC_Os08g36910 | -2.73467 | 3.95606 |  |  |  |
| LOC_Os03g49430 | -2.74125 | -2.5609 |  |  |  |
| LOC_Os04g51680 | -2.87595 | 2.18544 |  |  |  |
| LOC_Os01g15900 | -2.95103 | 2.55278 |  |  |  |
| LOC_Os01g14520 | -2.98043 | 4.00585 |  |  |  |
| LOC_Os10g41550 | -3.21924 | 2.14092 |  |  |  |
| LOC_Os03g22790 | -3.29437 | 2.4824 |  |  |  |
| LOC_Os02g08490 | -3.60104 | -2.30854 |  |  |  |
| LOC_Os02g12680 | -4.583 | 2.8493 |  |  |  |

**Supplementary Table 9:**  List of primer used for real time PCR

| **S.N.** | **SDEL** | **Putative Function** | **Primer Sequence** |
| --- | --- | --- | --- |
| 1 | LOC_Os03g02514_FP | hydrolase, alpha/beta fold family protein | TGCGGACGCTCTAGGATTA |
|  | LOC_Os03g02514_RP |  | CCTTTCTGGGATGTACTTGAGG |
|  |  |  |  |
| 2 | LOC_Os05g07870_FP | triose phosphate/phosphate translocator | GAGTATCACCTGTGACCCATTC |
|  | LOC_Os05g07870_RP |  | GCCAGTTCCAAGGGCATTA |
|  |  |  |  |
| 3 | LOC_Os08g33720_FP | lactate/malate dehydrogenase | CTGGCGACCCTGATGTTTAT |
|  | LOC_Os08g33720_RP |  | CACCGTTCTTTCCAAGCTTAAC |
|  |  |  |  |
| 4 | LOC_Os09g01960_FP | MYB family transcription factor | ACACCATCTTGTGTACG |
|  | LOC_Os09g01960_RP |  | AGCCGGACATGTAATTC |
|  |  |  |  |
| 5 | LOC_Os01g60020_FP | NAC domain transcription factor | CCGTACATGATGAACCT |
|  | LOC_Os01g60020_RP |  | ATTGTCTGCCACATCTT |
|  |  |  |  |
| 6 | LOC_Os06g48160_FP | glycosyl hydrolases family 16 | GTGGCGCTTGGCATTGT |
|  | LOC_Os06g48160_RP |  | CTCCGCGTCCTGGAAGAA |

**Supplementary Table 10:** Details of clustering between loci common between PB1+*Pi9* 24hpi and PB1+*Pi54* 72hpi.

| **Name of cluster** | **Locus id** | **log_2_(fold change)** | **log_2_(fold change)** |
| --- | --- | --- | --- |
| **Cluster a** |  | **PB1+*Pi9*** | **PB1+*Pi54*** |
|  | LOC_Os08g36910.1 | -2.73467 | 3.95606 |
|  | LOC_Os12g13800.1 | -2.05702 | 4.90091 |
|  | LOC_Os08g39730.1 | -2.3494 | 3.55501 |
|  | LOC_Os01g14520.1 | -2.98043 | 4.00585 |
|  | LOC_Os12g13810.1 | -2.05702 | 4.90091 |
|  | LOC_Os03g04100.1 | -2.07349 | 3.15463 |
|  |  |  |  |
| **Cluster b** |  | **PB1+*Pi9*** | **PB1+*Pi54*** |
|  | LOC_Os01g06876.1 | 3.64833 | 7.15203 |
|  | LOC_Os01g06836.1 | 3.74864 | 7.15203 |
|  | LOC_Os01g43460.2 | 2.81111 | 6.36797 |
|  | LOC_Os01g06882.1 | 3.64833 | 7.15203 |
|  | LOC_Os03g50960.1 | 4.74142 | 5.58052 |
|  |  |  |  |
| **Cluster c** |  | **PB1+*Pi9*** | **PB1+*Pi54*** |
|  | LOC_Os10g41550.1 | -3.21924 | 2.14092 |
|  | LOC_Os03g22790.1 | -3.29437 | 2.4824 |
|  | LOC_Os02g12680.1 | -4.583 | 2.8493 |
|  | LOC_Os01g15900.1 | -2.95103 | 2.55278 |
|  | LOC_Os01g04620.1 | -2.53338 | 2.68618 |
|  | LOC_Os04g51680.1 | -2.87595 | 2.18544 |
|  | LOC_Os03g13050.1 | -2.47531 | 2.01499 |
|  |  |  |  |
| **Cluster d** |  | **PB1+*Pi9*** | **PB1+*Pi54*** |
|  | LOC_Os07g22930.3 | 3.49943 | 3.07817 |
|  | LOC_Os08g04800.1 | 3.36148 | 3.9203 |
|  | LOC_Os06g40170.1 | 3.41174 | 4.28504 |
|  | LOC_Os08g14570.1 | 2.88613 | 3.39665 |
|  | LOC_Os08g14570.1 | 2.88613 | 3.39665 |
|  | LOC_Os05g50930.1 | 4.59257 | 3.73594 |
|  | LOC_Os02g57720.1 | 3.92771 | 3.4497 |
|  | LOC_Os04g48840.1 | 3.0812 | 3.38706 |
|  | LOC_Os01g16980.1 | 4.50624 | 4.14715 |
|  |  |  |  |
| **Cluster e** |  | **PB1+*Pi9*** | **PB1+*Pi54*** |
|  | LOC_Os05g12640.1 | 3.65176 | -2.20673 |
|  | LOC_Os12g39360.1 | 4.52345 | -2.09733 |
|  | LOC_Os12g39360.1 | 4.52345 | -2.09733 |
|  | LOC_Os01g12690.1 | 3.82672 | -2.33026 |
|  | LOC_Os05g12630.1 | 3.65176 | -2.20673 |
|  |  |  |  |
| **Cluster f** |  | **PB1+*Pi9*** | **PB1+*Pi54*** |
|  | LOC_Os01g64120.1 | 6.23682 | 2.73542 |
|  | LOC_Os07g03120.1 | 5.54212 | 2.5888 |
|  | LOC_Os01g55160.1 | 5.40103 | 2.96826 |
|  |  |  |  |
| **Cluster g** |  | **PB1+*Pi9*** | **PB1+*Pi54*** |
|  | LOC_Os02g01590.1 | 3.60012 | 2.08866 |
|  | LOC_Os02g11070.1 | 3.12395 | 2.01109 |
|  | LOC_Os12g18560.1 | 4.44624 | 2.01714 |
|  | LOC_Os09g26780.1 | 3.68759 | 2.95659 |
|  | LOC_Os04g45730.1 | 3.16643 | 2.33569 |
|  | LOC_Os10g42960.1 | 4.12387 | 2.74759 |
|  | LOC_Os10g41100.1 | 3.64876 | 2.01914 |
|  | LOC_Os01g03940.1 | 3.29724 | 2.14699 |
|  | LOC_Os01g17396.2 | 4.07819 | 2.16301 |
|  |  |  |  |
| **Cluster h** |  | **PB1+*Pi9*** | **PB1+*Pi54*** |
|  | LOC_Os02g52150.1 | -2.30324 | -2.31659 |
|  | LOC_Os02g08490.1 | -3.60104 | -2.30854 |
|  | LOC_Os03g49430.1 | -2.74125 | -2.5609 |
|  | LOC_Os04g31870.1 | -2.48067 | -2.18292 |
|  | LOC_Os03g01720.1 | -2.41145 | -2.49598 |
|  |  |  |  |
| **Cluster i** |  | **PB1+*Pi9*** | **PB1+*Pi54*** |
|  | LOC_Os01g71310.1 | 2.31911 | 3.31414 |
|  | LOC_Os01g18170.1 | 2.25407 | 3.26753 |
|  | LOC_Os09g28650.1 | 2.22341 | 4.76418 |
|  | LOC_Os04g32480.1 | 2.20383 | 4.64823 |
|  | LOC_Os02g13800.1 | 2.0439 | 4.12218 |
|  | LOC_Os08g28710.1 | 2.70315 | 3.67174 |
|  | LOC_Os05g47770.1 | 2.12191 | 3.23927 |
|  | LOC_Os05g47770.1 | 2.12191 | 3.23927 |
|  | LOC_Os09g38910.1 | 2.88872 | 3.89765 |
|  | LOC_Os01g62950.1 | 2.00652 | 4.79514 |
|  | LOC_Os06g49470.1 | 2.77283 | 4.15717 |
|  | LOC_Os01g60020.1 | 2.13745 | 3.30374 |
|  | LOC_Os11g41870.1 | 2.59256 | 4.68803 |
|  | LOC_Os04g59330.1 | 2.27877 | 4.11786 |
|  | LOC_Os05g30500.1 | 2.78046 | 4.48571 |
|  |  |  |  |
| **Cluster j** |  | **PB1+*Pi9*** | **PB1+*Pi54*** |
|  | LOC_Os08g33720.1 | 2.67669 | 2.57307 |
|  | LOC_Os08g33720.1 | 2.67669 | 2.57307 |
|  | LOC_Os04g51160.1 | 2.04805 | 2.70913 |
|  | LOC_Os01g22010.3 | 3.06646 | 2.35927 |
|  | LOC_Os04g33390.1 | 2.52726 | 2.29903 |
|  | LOC_Os04g27790.1 | 2.65387 | 2.55889 |
|  | LOC_Os08g38910.2 | 2.28348 | 2.68564 |
|  | LOC_Os09g20090.1 | 2.14264 | 2.87217 |
|  | LOC_Os02g52780.1 | 2.48384 | 3.07346 |
|  | LOC_Os07g40290.1 | 2.31828 | 2.3902 |
|  | LOC_Os09g02710.1 | 2.59983 | 2.2522 |
|  | LOC_Os09g02710.1 | 2.59983 | 2.2522 |
|  | LOC_Os06g11210.1 | 3.04133 | 2.69534 |
|  | LOC_Os06g11240.1 | 2.39485 | 2.21811 |
|  | LOC_Os07g42370.1 | 2.14094 | 2.4233 |
|  | LOC_Os01g44050.1 | 2.86709 | 2.26886 |
|  | LOC_Os10g39680.1 | 2.57069 | 2.30028 |
|  | LOC_Os01g64110.1 | 2.67463 | 2.5674 |
|  | LOC_Os01g53090.1 | 2.37497 | 2.70327 |
|  | LOC_Os07g42370.1 | 2.14094 | 2.4233 |
|  | LOC_Os04g46970.1 | 2.04642 | 2.7518 |
|  | LOC_Os04g41970.1 | 2.76594 | 2.93546 |
|  | LOC_Os01g37750.1 | 2.78035 | 2.7241 |
|  | LOC_Os09g04050.1 | 2.13754 | 2.97178 |
|  | LOC_Os05g05640.1 | 2.29132 | 2.847 |
|  | LOC_Os07g38290.1 | 2.09337 | 2.40337 |
|  | LOC_Os03g59670.1 | 2.18808 | 2.8639 |
|  | LOC_Os05g37190.1 | 2.36686 | 2.82924 |
|  | LOC_Os09g01960.1 | 2.34221 | 2.23513 |
|  | LOC_Os02g48320.3 | 2.16612 | 2.16287 |
|  | LOC_Os07g02780.1 | 2.20892 | 2.30848 |
|  | LOC_Os03g50810.1 | 2.18096 | 2.68995 |
|  | LOC_Os09g39190.1 | 2.10553 | 3.07074 |
|  | LOC_Os06g12560.1 | 2.33276 | 2.28592 |
|  | LOC_Os02g54060.1 | 2.26919 | 2.107 |
|  | LOC_Os09g38850.1 | 2.01882 | 2.13427 |
|  | LOC_Os07g04560.1 | 2.46817 | 2.19185 |
|  | LOC_Os01g11160.1 | 2.45181 | 2.33773 |
|  | LOC_Os05g07870.1 | 2.68396 | 2.69092 |
|  | LOC_Os04g33060.1 | 2.50484 | 2.66517 |
|  | LOC_Os09g06464.1 | 2.24457 | 2.34498 |
|  | LOC_Os03g02514.1 | 2.69383 | 2.95308 |
|  | LOC_Os09g32100.1 | 2.82234 | 2.05787 |
|  | LOC_Os08g07620.1 | 2.61824 | 2.05912 |
|  | LOC_Os01g72610.1 | 2.57008 | 2.04623 |
|  | LOC_Os06g36070.1 | 2.95561 | 2.52983 |
|  | LOC_Os02g11870.1 | 2.39326 | 2.52687 |
|  | LOC_Os04g49950.1 | 2.49543 | 3.21126 |
|  | LOC_Os10g37570.1 | 3.02655 | 2.9215 |
|  | LOC_Os02g11859.1 | 2.21429 | 2.2067 |
|  | LOC_Os09g25720.1 | 2.96377 | 2.20358 |
|  | LOC_Os03g01990.1 | 2.0437 | 2.19046 |
|  | LOC_Os02g43540.1 | 2.82892 | 2.26003 |
|  | LOC_Os08g31850.1 | 2.46943 | 2.47784 |
|  | LOC_Os01g69960.1 | 2.17596 | 2.03401 |

**Supplementary Table 11**: Details of co-expression network formed by unique SDEL common between PB1+*Pi9* 24hpi and PB1+*Pi54* 72hpi

| **#node1** | **node2** | **node1_external_id** | **node2_external_id** | **neighborhood_on_chromosome** | **gene_fusion** | **phylogenetic_cooccurrence** | **homology** | **coexpression** | **experimentally_determined_interaction** | **database_annotated** | **automated_textmining** | **combined_score** |
| --- | --- | --- | --- | --- | --- | --- | --- | --- | --- | --- | --- | --- |
| OsJ_05853 | 4328676 | 39947.LOC_Os02g11859.1 | 39947.LOC_Os02g11070.1 | 0 | 0 | 0 | 0 | 0.935 | 0 | 0 | 0 | 0.935 |
| 4349417 | 4345814 | 39947.LOC_Os10g41550.1 | 39947.LOC_Os08g36910.1 | 0 | 0 | 0 | 0 | 0 | 0 | 0.9 | 0.098 | 0.905 |
| 4345814 | 4343010 | 39947.LOC_Os08g36910.1 | 39947.LOC_Os07g22930.2 | 0.576 | 0 | 0 | 0 | 0 | 0.067 | 0 | 0.569 | 0.814 |
| 4330786 | 4328515 | 39947.LOC_Os02g52150.1 | 39947.LOC_Os02g08490.1 | 0.214 | 0 | 0 | 0 | 0.456 | 0.107 | 0 | 0.548 | 0.804 |
| 4328676 | 4327485 | 39947.LOC_Os02g11070.1 | 39947.LOC_Os01g60020.1 | 0 | 0 | 0 | 0 | 0.781 | 0 | 0 | 0 | 0.781 |
| OsJ_17288 | 4335739 | 39947.LOC_Os05g07870.1 | 39947.LOC_Os04g33060.1 | 0 | 0 | 0 | 0 | 0.78 | 0 | 0 | 0 | 0.78 |
| 4343010 | 4335739 | 39947.LOC_Os07g22930.2 | 39947.LOC_Os04g33060.1 | 0 | 0 | 0 | 0 | 0.778 | 0 | 0 | 0 | 0.778 |
| 4347164 | 4343899 | 39947.LOC_Os09g26780.1 | 39947.LOC_Os07g42370.1 | 0 | 0 | 0 | 0.837 | 0.75 | 0 | 0 | 0.552 | 0.771 |
| OsJ_04149 | OsJ_02706 | 39947.LOC_Os01g64120.1 | 39947.LOC_Os01g44050.1 | 0 | 0 | 0 | 0 | 0.75 | 0 | 0 | 0 | 0.75 |
| 4345047 | OsJ_02706 | 39947.LOC_Os08g14570.1 | 39947.LOC_Os01g44050.1 | 0.366 | 0 | 0 | 0 | 0.158 | 0 | 0 | 0.551 | 0.739 |
| 4330925 | 4328515 | 39947.LOC_Os02g54060.1 | 39947.LOC_Os02g08490.1 | 0.063 | 0 | 0 | 0 | 0.261 | 0 | 0 | 0.613 | 0.708 |
| 4345047 | 4331400 | 39947.LOC_Os08g14570.1 | 39947.LOC_Os03g02514.1 | 0 | 0 | 0 | 0 | 0.705 | 0 | 0 | 0 | 0.705 |
| 4345414 | 4335698 | 39947.LOC_Os08g28710.1 | 39947.LOC_Os04g32480.1 | 0 | 0 | 0 | 0 | 0.676 | 0 | 0 | 0 | 0.676 |
| 4343899 | 4335698 | 39947.LOC_Os07g42370.1 | 39947.LOC_Os04g32480.1 | 0 | 0 | 0 | 0 | 0.291 | 0 | 0 | 0.549 | 0.666 |
| 4345047 | OsJ_04149 | 39947.LOC_Os08g14570.1 | 39947.LOC_Os01g64120.1 | 0 | 0 | 0 | 0 | 0 | 0.25 | 0 | 0.566 | 0.66 |
| OsJ_05853 | 4327485 | 39947.LOC_Os02g11859.1 | 39947.LOC_Os01g60020.1 | 0 | 0 | 0 | 0 | 0.651 | 0 | 0 | 0 | 0.651 |
| 4345414 | OsJ_05853 | 39947.LOC_Os08g28710.1 | 39947.LOC_Os02g11859.1 | 0 | 0 | 0 | 0 | 0.65 | 0 | 0 | 0 | 0.65 |
| 4346474 | 4339759 | 39947.LOC_Os09g06464.1 | 39947.LOC_Os05g50930.1 | 0 | 0 | 0 | 0 | 0.642 | 0 | 0 | 0 | 0.642 |
| 4345414 | 4327485 | 39947.LOC_Os08g28710.1 | 39947.LOC_Os01g60020.1 | 0 | 0 | 0 | 0 | 0.641 | 0 | 0 | 0 | 0.641 |
| 4339759 | 4335739 | 39947.LOC_Os05g50930.1 | 39947.LOC_Os04g33060.1 | 0 | 0 | 0 | 0 | 0.635 | 0 | 0 | 0 | 0.635 |
| 4349376 | OsJ_01322 | 39947.LOC_Os10g41100.1 | 39947.LOC_Os01g17396.2 | 0 | 0 | 0 | 0 | 0.608 | 0 | 0 | 0 | 0.608 |
| 4349376 | 4339759 | 39947.LOC_Os10g41100.1 | 39947.LOC_Os05g50930.1 | 0 | 0 | 0 | 0 | 0.592 | 0 | 0 | 0 | 0.592 |
| 4328696 | OsJ_05853 | 39947.LOC_Os02g11870.1 | 39947.LOC_Os02g11859.1 | 0 | 0 | 0 | 0 | 0.587 | 0 | 0 | 0 | 0.587 |
| 4349376 | 4335739 | 39947.LOC_Os10g41100.1 | 39947.LOC_Os04g33060.1 | 0 | 0 | 0 | 0 | 0.581 | 0 | 0 | 0 | 0.581 |
| 4346474 | 4346379 | 39947.LOC_Os09g06464.1 | 39947.LOC_Os09g01960.1 | 0 | 0 | 0 | 0 | 0 | 0.064 | 0 | 0.549 | 0.559 |
| 4339759 | 4330925 | 39947.LOC_Os05g50930.1 | 39947.LOC_Os02g54060.1 | 0.083 | 0 | 0 | 0 | 0 | 0 | 0 | 0.529 | 0.549 |
| 4347857 | 4341467 | 39947.LOC_Os09g38850.1 | 39947.LOC_Os06g40170.1 | 0 | 0 | 0 | 0 | 0.111 | 0.067 | 0 | 0.475 | 0.526 |
| 4343010 | 4341467 | 39947.LOC_Os07g22930.2 | 39947.LOC_Os06g40170.1 | 0 | 0 | 0 | 0 | 0 | 0 | 0 | 0.513 | 0.513 |
| 4345814 | 4341467 | 39947.LOC_Os08g36910.1 | 39947.LOC_Os06g40170.1 | 0 | 0 | 0 | 0 | 0 | 0 | 0 | 0.51 | 0.51 |
| 4340486 | 4327485 | 39947.LOC_Os06g11240.1 | 39947.LOC_Os01g60020.1 | 0 | 0 | 0 | 0 | 0 | 0 | 0 | 0.499 | 0.499 |
| 4339759 | OsJ_01322 | 39947.LOC_Os05g50930.1 | 39947.LOC_Os01g17396.2 | 0 | 0 | 0 | 0 | 0.496 | 0 | 0 | 0 | 0.496 |
| 4347859 | 4341467 | 39947.LOC_Os09g38910.1 | 39947.LOC_Os06g40170.1 | 0 | 0 | 0 | 0 | 0 | 0.067 | 0 | 0.475 | 0.489 |
| 4328696 | 4328676 | 39947.LOC_Os02g11870.1 | 39947.LOC_Os02g11070.1 | 0 | 0 | 0 | 0 | 0.473 | 0 | 0 | 0 | 0.473 |
| 4335698 | 4328676 | 39947.LOC_Os04g32480.1 | 39947.LOC_Os02g11070.1 | 0 | 0 | 0 | 0 | 0.466 | 0 | 0 | 0 | 0.465 |
| 4339759 | 4328515 | 39947.LOC_Os05g50930.1 | 39947.LOC_Os02g08490.1 | 0 | 0 | 0 | 0 | 0 | 0.19 | 0 | 0.337 | 0.44 |
| 4345414 | 4328676 | 39947.LOC_Os08g28710.1 | 39947.LOC_Os02g11070.1 | 0 | 0 | 0 | 0 | 0.435 | 0 | 0 | 0 | 0.435 |
| 4336875 | OsJ_05853 | 39947.LOC_Os04g51160.1 | 39947.LOC_Os02g11859.1 | 0 | 0 | 0 | 0 | 0.426 | 0 | 0 | 0 | 0.426 |
| 4349376 | 4346474 | 39947.LOC_Os10g41100.1 | 39947.LOC_Os09g06464.1 | 0 | 0 | 0 | 0.698 | 0.417 | 0 | 0 | 0 | 0.416 |
| 4345414 | 4338934 | 39947.LOC_Os08g28710.1 | 39947.LOC_Os05g37190.1 | 0 | 0 | 0 | 0 | 0.364 | 0.073 | 0 | 0.08 | 0.41 |
| 4335698 | 4327485 | 39947.LOC_Os04g32480.1 | 39947.LOC_Os01g60020.1 | 0 | 0 | 0 | 0 | 0.404 | 0 | 0 | 0 | 0.404 |
| 4347164 | 4345657 | 39947.LOC_Os09g26780.1 | 39947.LOC_Os08g33720.1 | 0 | 0 | 0 | 0 | 0.402 | 0 | 0 | 0 | 0.402 |
| 4343010 | 4339759 | 39947.LOC_Os07g22930.2 | 39947.LOC_Os05g50930.1 | 0 | 0 | 0 | 0 | 0.375 | 0 | 0 | 0.08 | 0.4 |
| 4325538 | 4325684 | 39947.LOC_Os01g11160.1 | 39947.LOC_Os01g04620.1 | 0.362 | 0 | 0 | 0 | 0 | 0 | 0 | 0.099 | 0.4 |

**Supplementary Table 12:** Pathway found in co-expression network of proteins corresponding to the significant (log_2_fold change ≥ 2) loci common between resistant NILs PB1+*Pi9* 24hpi and PB1+*Pi54* 72hpi

| **Pathway description** | **Observed gene count** | **FDR** |
| --- | --- | --- |
| oxylipin biosynthetic process | 3 | 0.0266 |
| molecular_function | 16 | 0.0446 |
| O-methyltransferase activity | 2 | 0.0446 |
| Plant hormone signal transduction | 5 | 0.00348 |
